# Multimaterial 4D Printing with Tailorable Shape Memory Polymers

**DOI:** 10.1038/srep31110

**Published:** 2016-08-08

**Authors:** Qi Ge, Amir Hosein Sakhaei, Howon Lee, Conner K. Dunn, Nicholas X. Fang, Martin L. Dunn

**Affiliations:** 1 Digital Manufacturing and Design Center, Singapore University of Technology and Design, Singapore; 2 Department of Mechanical Engineering, Massachusetts Institute of Technology, Cambridge, MA, 02139, USA; 3 The George W. Woodruff School of Mechanical Engineering, Georgia Institute of Technology, Atlanta, GA, 30332, USA

## Abstract

We present a new 4D printing approach that can create high resolution (up to a few microns), multimaterial shape memory polymer (SMP) architectures. The approach is based on high resolution projection microstereolithography (P*μ*SL) and uses a family of photo-curable methacrylate based copolymer networks. We designed the constituents and compositions to exhibit desired thermomechanical behavior (including rubbery modulus, glass transition temperature and failure strain which is more than 300% and larger than any existing printable materials) to enable controlled shape memory behavior. We used a high resolution, high contrast digital micro display to ensure high resolution of photo-curing methacrylate based SMPs that requires higher exposure energy than more common acrylate based polymers. An automated material exchange process enables the manufacture of 3D composite architectures from multiple photo-curable SMPs. In order to understand the behavior of the 3D composite microarchitectures, we carry out high fidelity computational simulations of their complex nonlinear, time-dependent behavior and study important design considerations including local deformation, shape fixity and free recovery rate. Simulations are in good agreement with experiments for a series of single and multimaterial components and can be used to facilitate the design of SMP 3D structures.

Three-dimension (3D) printing technology allows the creation of complex geometries with precisely prescribed microarchitectures that enable new functionality or improved and even optimal performance. While 3D printing has largely emphasized manufacturing with a single material, recent advances in multimaterial printing enable the creation of heterogeneous structures or composites that have myriad scientific and technological applications^1^,^2^,^3^,^4^,^5^. Commercial printing systems with these capabilities have been used in many innovative applications, but are limited because their development has largely proceeded with objectives of creating printed components with reliable mechanical properties, and applications have generally emphasized linear elastic behavior with small deformations where the innovation arises from the sophisticated geometry. Independently, soft active materials (SAMs) as a class of emerging materials with the capability of exhibiting large elastic deformation in response to environmental stimuli such as heat^6^,^7^, light^8^,^9^, and electricity^10^,^11^, are enabling the creation of functional active components. SAMs including shape memory polymers (SMPs), hydrogels, dielectric elastomers have been used to fabricate biomedical devices^7^,^12^,^13^, wearable devices^14^,^15^, artificial muscles^10^,^11^ and other smart products^16^,^17^,^18^. However, applications of SAMs are limited by the current manufacturing approaches which constrain active structures and devices to simple geometries, often created with a single material, and they have yet to broadly exploit the potential of tailored microarchitectures.

This picture is changing as 3D printing and SAMs are being integrated. The most notable example is the recently developed “4D printing” technology^2^,^3^ in which the printed 3D structures are able to actively transform configurations over time in response to environmental stimuli. There are two types of SAMs mainly used to realize 4D printing: hydrogels that swell when solvent molecules diffuse into polymer network and shape memory polymers (SMPs) that are capable of fixing temporary shapes and recovering to the permanent shape upon heating. The examples of the hydrogel-based 4D printing include complex self-evolving structures actuated by multilayer joints^5^, active valves made of thermally sensitive hydrogel^19^, pattern transformation realized by heat-shrinkable polymer^20^, and biominic 4D printing achieved by anisotropic hydrogel composites with cellulose fibrils^21^. However, the low modulus of hydrogels ranging from ∼kPa to ∼100 kPa^19^,^21^, and the solvent diffusion based slow response rates in the time scale of a few ten minutes, hours, and even days^5^,^21^,^22^ make the hydrogel based 4D printing not suitable for structural and actuation applications. Compared to hydrogels, SMPs have higher modulus ranging ∼MPa to ∼GPa^7^,^23^ and faster response rates (in the scale of seconds to minutes depending on actuation temperature)^24^,^25^. The examples of the SMP based 4D printing include printed active composites where precisely prescribed SMP fibers were used to activate the complex shape change^2^,^3^, sequential self-folding structured where SMP hinges with different responding rates were deliberately placed at different positions^4^, and multi-shape active composites where two SMP fibers with different glass transition temperatures^26^. To date, the SMP based 4D printing were mainly realized by commercial Polyjet 3D printer (Stratasys, Objet) which create materials with properties ranging between rigid and elastomeric by mixing the two base resins. The fact that users are not allowed to freely tune the thermomechanical properties beyond the realm of available resins impedes this 4D printing technology to advance to a wider range of applications. For example, the capability of 4D printed actuation is limited as the printed digital materials break at 10–25% 27; the printed structures could not be used in high temperature applications as the highest glass transition temperature of the available resin is about ∼70 °C^4^. In addition, this technology is not suitable for microscale applications as the lateral printing resolution is up to 200 *μ*m inherently limited by the Polyjet printing method^28^.

In this paper we report a new approach that enables high resolution multimaterial 4D printing by printing highly tailorable SMPs on a projection microstereolithography (P*μ*SL) based additive manufacturing system. We synthesize photo-curable methacrylate based copolymer networks using commercially-available materials. By tuning the material constituents and compositions, the flexibility of the methacrylate based copolymer networks enables highly tailorable SMP thermomechanical properties including rubbery modulus (from ∼MPa to ∼100 MPa), glass transition temperature (from ∼−50 °C to ∼180 °C), and the failure strain (up to ∼300%). Methacrylate based SMPs with different constituents or compositions form strong interface bonds with each other and enable fabrication of 3D structures made of multiple SMPs that can exhibit new functionality resulting from their dynamic thermomechanical properties. The P*μ*SL based additive manufacturing system with high lateral resolution up to ∼1 *μ*m exploits a digital micro-display device as a dynamic photo mask to dynamically generate and reconfigure light patterns, which then converts liquid monomer resin into solid *via* photo-polymerization in a layer-by-layer fashion^29^,^30^,^31^. A high resolution, high contrast digital micro-display device ensures high resolution structure made of methacrylate based SMPs that require higher exposure energy than acrylate based polymers which have been frequently used for 3D printing but do not have SM effect. Multimaterial manufacturing is achieved *via* an automatic material exchanging mechanism integrated into the P*μ*SL additive manufacturing system. In addition, a highly fidelity computational tool based on the understanding of the shape memory behavior is used to facilitate the design of SMP 3D structures by simulating important design considerations including local deformation, shape fixity and free recovery rate. We believe this novel approach will translate the SMP based 4D printing into a wide variety of practical applications, including biomedical devices^12^,^13^,^32^,^33^, deployable aerospace structures^34^,^35^, shopping bags^36^,^37^, and shape changing photovoltaic solar cells^38^,^39^.

## Results

### Multimaterial additive manufacturing system

**Figure 1:**
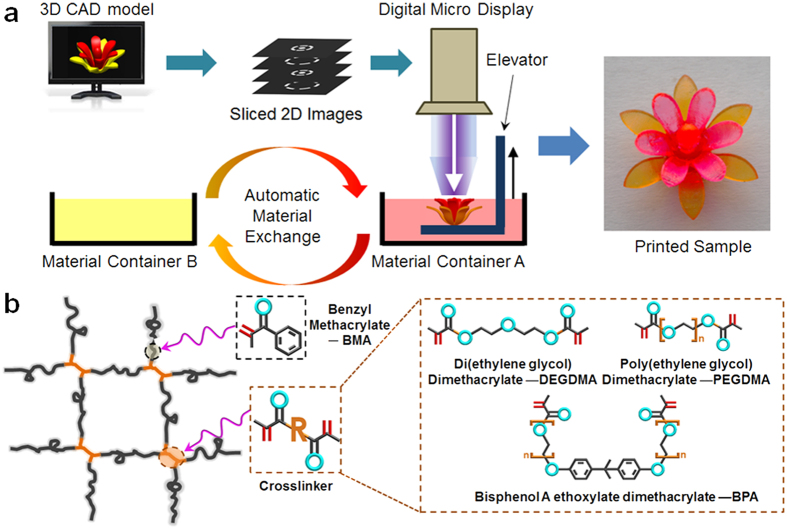
Schematics of multimaterial additive manufacture system. (**a**) A workflow illustrates the process of fabricating a multimaterial structure based on P*μ*SL (**b**) Photo-curable shape memory polymer network is constructed by mono-functional monomer, Benzyl methacrylate (BMA) as linear chain builder (LCB), and multi-functional oligomers, Poly (ethylene glycol) dimethacrylate (PEGDMA), Bisphenol A ethoxylate dimethacrylate (BPA), and Di(ethylene glycol) dimethacrylate (DEGDMA) as crosslinkers.

We fabricate high resolution multimaterial shape memory structures on an additive manufacturing apparatus based on projection microstereolithography (P*μ*SL)^29^,^30^,^31^. As shown schematically in Fig. 1a, a computer aid design (CAD) model is first sliced into a series of closely spaced horizontal two-dimension (2D) digital images. Then, these 2D images are transmitted to a digital micro display which works as a dynamic photo-mask^30^. Ultraviolet (UV) light produced from a light emitting diode (LED) array is spatially modulated with the patterns of the corresponding 2D images, and illuminated onto the surface of photo-curable polymer solution. Once the material in the exposed area is solidified to form a layer, the substrate on which the fabricated structure rests is lowered by a translational stage, followed by projection of the next image to polymerize a new layer on top of the preceding one. This process proceeds iteratively until the entire structure is fabricated. In the current setup, the projection area is about 3.2 cm × 2.4 cm resulting in a pixel size of ∼30 *μ*m × 30 *μ*m. The lateral resolution can be further improved up to as high as ∼1 *μ*m if a projection lens with high optical magnification is used^29^. The step-and-repeat method can be employed to extend printing area without compromising lateral resolution^30^. Multimaterial fabrication is enabled by automating polymer solution exchange during the printing process. Although many efforts^40^,^41^,^42^ have been made to develop multimaterial fabrication systems by adding the automated polymer solution exchanging mechanisms into the “top-down” fabrication system (as shown schematically in Fig. 1a) where the modulated UV light projected downwards to the polymer resin, the multimaterial fabrication system developed based on the “bottom-up” approach making the depth of transparent polymer solution containers independent of the part height helps to significantly reduce material contamination and improve efficiency of material use^43^, but requires precise control of the oxygen concentration to separate the printed parts from the transparent polymer solution containers without damaging them^43^,^44^.

We fabricate shape memory structures using photo-curable methacrylate copolymers that form polymer networks *via* free radical photo-polymerization^45^,^46^,^47^. To understand the thermomechanical properties and shape memory (SM) effects of the materials and structures, we prepared polymer resins by using a mono-functional monomer, Benzyl methacrylate (BMA) as linear chain builder (LCB), and difunctional oligomers, Poly (ethylene glycol) dimethacrylate (PEGDMA), Bisphenol A ethoxylate dimethacrylate (BPA), and Di(ethylene glycol) dimethacrylate (DEGDMA) as crosslinkers that connect the linear chains to form a cross-linked network (shown in Fig. 1b). Details about polymer resin preparations can be founded in Methods. More selections of LCBs and crosslinkers are suggested by Safranski and Gall^48^.

### Experimental Characterization

**Figure 2:**
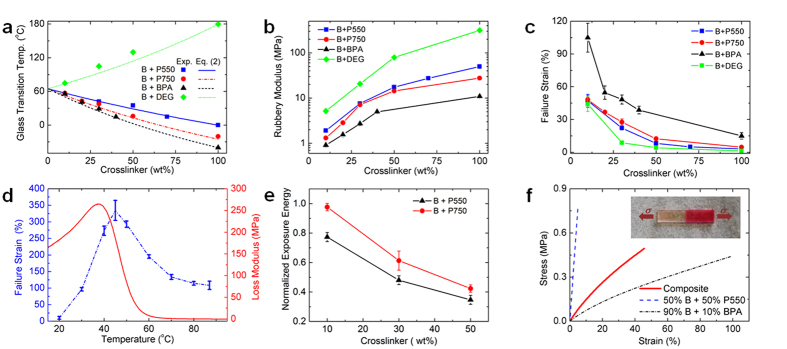
Experimental characterization of methacrylate SMP networks. Highly tailorable glass transition temperature (**a**), rubbery modulus (**b**), and failure strain (**c**) are controlled by either changing the mixing LCB/crosslinker ratio or using different crosslinkers. (**d**) The temperature effect on the failure strain of the SMP consisting of 90% BMA and 10% BPA. (**e**) The normalized exposure energy to cure a thin layer varies with the crosslinker concentration as well as the molecular weight of crosslinker. (**f**) The investigation on the interface bonding of a printed composite with two components arranged in series (inset).

The photo-curable methacrylate networks provide high tailorability of thermomechanical properties of printed SMPs. Among them, the glass transition temperature (*T*_*g*_), the rubbery modulus (*E*_*r*_) and the failure strain (*ε*_*f*_) are the most critical properties for the design of active components as they dictate the shape recovery temperature and rate, constrained recovery stress, and capability of shape change and/or actuation, respectively^24^,^48^,^49^,^50^,^51^. Figure 2a–c demonstrates that these thermomechanical properties can be tailored over wide ranges and still printed, by either controlling the concentration of crosslinker or using different crosslinkers. In Fig. 2a, for instance, for the copolymer network system consisting of BMA and crosslinker PEGDMA with molecular weight of 550 g/mole (denoted as B + P550), the *T*_*g*_ starts from ∼65 °C where the copolymer network system consists of pure BMA, and then decreases with increase in the crosslinker concentration (See Supplementary Materials S1.1 I and Fig. S1a). The *T*_*g*_ of the copolymer networks consisting of the crosslinker PEGDMA with molecular weight of 750 g/mole (denoted as B + P750) and the crosslinker BPA with molecular weight of 1700 g/mole (denoted as B + BPA) follows the same trend of the B + P550 copolymer networks (Fig. S1b,c), while the *T*_*g*_ increases with increase in the crosslinker concentration of the copolymer network consisting of BMA and DEGDMA (denoted as B + DEG, Fig. S1d). The Couchman equation^52^ can be used to guide material design with a desired *T*_*g*_ by mixing the LCB and crosslinker with prescribed ratios: 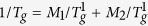. Here, 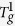 and 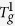 are the glass transition temperatures of the respective pure-components, and *M*_1_ and *M*_2_ are the corresponding mass fractions. In Fig. 2a, using the current LCB monomer, namely BMA, and crosslinkers, namely PEGDMA, BPA and DEGDMA allows us to adjust *T*_*g*_ from ∼−50 °C to ∼180 °C, while more flexibility can be obtained by choosing different LCB monomers and crosslinkers^48^ or even mixing more than one LCB monomers and crosslinkers to prepare the polymer resin^53^.

Figure 2b shows that the rubbery modulus *E*_*r*_ of the copolymer networks increases with increase in crosslinker concentration (see Supplementary Materials S1.2, Fig. S2 and Table S1), as expected from entropic elasticity^54^, *E*_*r*_ = (3*ρRT*)/*M*_*c*_; here, *R* is the gas constant, *T* is absolute temperature, *ρ* is polymer density, and *M*_*c*_ is average molecular weight between crosslinks. The ratio *ρ*/*M*_*c*_ is the crosslinking density of the polymer network which is affected by crosslinker concentration as well as the molecular weight of the crosslinker. Comparing the four network systems, the B + DEG network has the highest rubbery modulus at the same mass fraction of crosslinker, as the highest molar weight of DEGDMA leads to the highest crosslinking density (See Supplementary Materials S1.3 and Table S2) and the highest *E*_*r*_.

The effect of crosslinker on failure strain *ε_f_* is shown in Fig. 2c; these results were obtained from uniaxial tensile tests at temperatures 30 °C above each sample’s *T*_*g*_ where sample stays the rubbery state to eliminate the effect of viscoelasticity (see Supplementary Materials S1.2, Fig. S2 and Table S1). The results shown in Fig. 2c suggest that in the SMP system consisting of the same LCB and crosslinker, the lower crosslinker concentration gives higher stretchability. Figure 2c also shows that the copolymer network system formed with a higher molecular weight crosslinker has higher stretchability. For example, for the copolymer systems consisting of 10% crosslinkers, the stretchability can be increased from ∼45% to ∼100% at 30 °C above sample’s *T*_*g*_ by increase the molecular weight from 242.3 g/mol (DEGDMA) to 1700 g/mol (BPA) (See Supplementary Materials S1.2, Fig. S2 and Table S1). Figure 2d shows the temperature effect on the failure strain of an SMP sample consisting of 90% BMP and 10% BPA (See Supplementary Materials S1.4 and Fig. S3). The stretchability of this copolymer network is increased by decreasing the stretching temperatures, and finally reaches the maximum of ∼330% at 40 °C where the temperature is close to the peak of the loss modulus indicating the highest energy dissipation. A more stretchable network can be achieved by further reducing the crosslinker concentration of BPA or replacing BPA with a crosslinker having higher molecular weight.

Not only does the chemical composition affect the thermomechanical properties of the printed SMP systems, it also affects the photo-polymerization kinetics that determines the build rate during manufacturing. As shown in Fig. 2e, at a given UV light intensity, less exposure energy (time) is required to cure a layer of the same thickness when the crosslinker concentration increases (See Supplementary Materials S1.5 and Fig. S4a). This is mainly attributed to the reaction diffusion-controlled termination during the polymerization of the methacrylate based copolymer system^55^,^56^,^57^. With more crosslinker, the crosslinking density of the polymer increases, which limits the propagation of free radicals that would otherwise reach each other to terminate the polymerization^57^. Figure 2e also shows that the copolymer network consisting of a lower molecular weight crosslinker (P550) requires less exposure energy (time) to polymerize a same thickness layer. This is primarily because the low molecular weight crosslinker (P550) contains more unreacted double bonds per unit mass than the high molecular weight crosslinker (P750) does. In addition, the increase of photo initiator reduces the exposure energy (time) to cure a same thickness layer (See Supplementary Materials S1.5 and Fig. S4b). Moreover, it is necessary to note that under the same condition methacrylate based SMP has comparatively lower reactivity^57^ than those acrylate based materials such as poly(ethylene glycol) diacrylate (PEGDA) and hexanediol diacrylate which have been frequently used to print 3D structure^29^,^30^,^31^,^44^,^58^,^59^. This comparatively slow but conversion-dependent^57^ photo-polymerization kinetics makes the methacrylate based SMPs require higher (longer) exposure energy (time) to cure a layer of the same thickness than the acrylate based materials (See Supplementary Materials S1.5 and Fig. S4c). Therefore, a high contrast digital micro display with moderate intensity of UV light is needed to avoid any unwanted curing on the unintended parts (Details about the digital micro display are described in Materials).

In a printed component that consists of more than one material, the interface bonding between them significantly impacts the mechanical performance of the composite. In Fig. 2f, we investigated the interfacial bonding by uniaxially stretching a composite with two components arranged in series (Component A: 50% B + 50% P550 with *T*_*g*_ = 31 °C and Component B: 90% B + 10% BPA *T*_*g*_ = 56 °C) at a temperature 30 °C higher than *T*_*g*_ of Component B, where the both components are in their rubbery state (See Supplementary Materials S1.6 and Fig. S5). The fact that the composite breaks at Component A which has a lower failure strain rather than at the interface indicates a strong interface bonding. The comparison of uniaxial tensile tests between the composite and the two components in Fig. 2e reveals that Components A and B form a strong covalently boned interface through which the composite transfers stress completely between the two components. Generally speaking, this strong interface bonding forms between the methacrylate based SMPs made of different compositions and constitutes.

### Shape Memory Behavior

**Figure 3:**
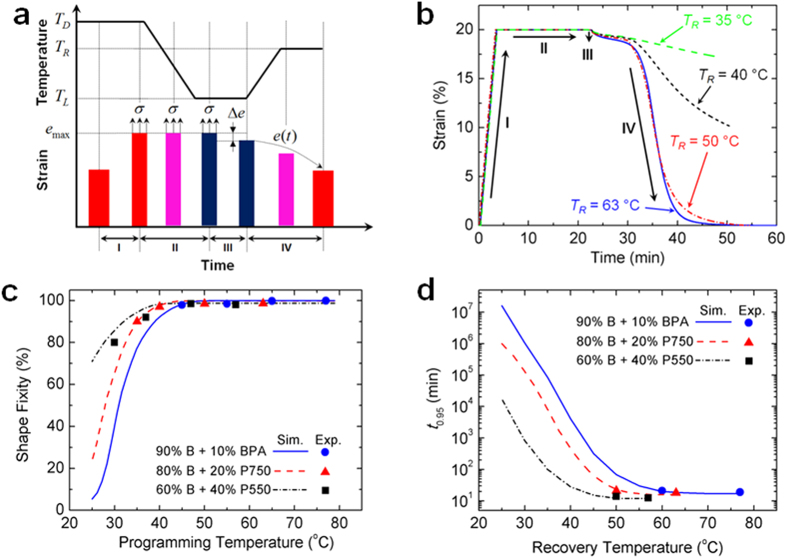
SM behavior of the (meth)acrylate based copolymer SMP network. (**a**) The SM behavior has been investigated by following a typical SM cycle: at Step I, a sample is deformed by *e*_max_ at a programming temperature *T*_*D*_; at Step II, the temperature is decreased from *T*_*D*_ to *T*_*L*_ while keeping the sample deformed by *e*_max_; at Step III, after unloading, there is a deformation bounce back Δ*e*; at Step IV, the free recovery is performed by heating the sample to a recovery temperature *T*_*R*_. (**b**) The representative SMP strain-time curves achieved by stretching a SMP sample (80% BMA and 20% P750) at 63 °C, unloading at 25 °C, and heating to 63 °C, 50 °C, 40 °C and 35 °C, respectively. (**c**) Shape fixity as a function of programming temperature. (**d**) Shape recovery time (*t*_0.95_) as a function of recovery temperature.

Two key attributes of SMPs are their ability to fix a temporary programmed shape (*fixity*) and to subsequently recover the original shape upon activation by a stimulus (*recovery*). Figure 3a shows a typical temperature-strain-time shape memory (SM) cycle that we used to investigate the fixity and recovery of SMP samples synthesized from different LCBs and crosslinkers that result in different *T*_*g*_ s. Figure 3b presents representative strain-time curves of a SMP strip sample made of 80% BMA + 20% P750. The SMP was first stretched to a target strain *e*_max_ (20%) with a constant loading rate *e* (0.001 s^−1^) at a programming temperature *T*_*D*_ (63 °C), and then the temperature was decreased to a *T*_*L*_ (*T*_*L*_ = 25 °C) with a cooling rate 2.5 °C/min. Once *T*_*L*_ was reached, the specimen was held for 2 minutes, and then the tensile force was removed. In the free recovery step, the temperature was increased to a recovery temperature *T*_*R*_ (in Fig. 3b, *T*_*R*_ = 35 °C, 40 °C, 50 °C and 60 °C, respectively) at the same rate of cooling and subsequently stabilized for another 20 min. (Details about the SM behavior testing are presented in Supplementary Material in S2.1 and Fig. S6).

As shown in Fig. 3a,b, we use the small strain bounce back, Δ*e*, of the SMP after unloading to define the shape fixity, i.e., *R*_*f*_ = (*e*_max_ − Δ*e*)/*e*_max_. Figure 3c shows that the shape fixity is a function of the programming temperature *T*_*D*_ (Details about *T*_*D*_ are listed in Supplementary Material Table S4): the SMP keeps a high shape fixity (>90%) when *T*_*D*_ is above or near the SMP’s *T*_*g*_, and the shape fixity starts to drop dramatically when *T*_*D*_ is 20 °C lower than the SMP’s *T*_g_. The phenomenon that the shape fixity is a function of *T*_*D*_ agrees the previous study^24^, and can be simulated by the recently developed multi-branch model which consists of an equilibrium branch corresponding to entropic elasticity and several thermoviscoelastic nonequilibrium branches to represent the multiple relaxation processes of the polymer^24^,^60^ (Details about the multi-branch model are presented in Supplementary Material S2.2–2.3). The model predictions agree with the experiments well and provide underlying understanding of the effect of the programming temperature *T*_*D*_ on the shape fixity *R*_*f*_ (Details about model predictions are presented in Supplementary Material S2.4 and Figure S9). When *T*_*D*_ is higher or near the *T*_*g*_, an SMP has a high shape fixity *R*_*f*_, as the time requires to relax all the nonequilibrium stresses is shorter than the time used for loading at *T*_*D*_ and cooling to *T*_*L*_. When *T*_*D*_ is decreased to a lower temperature the shape fixity *R*_*f*_ decreases as the nonequilibrium stresses do not have sufficient time to relax. For example, in Fig. 3c, the simulation indicates that for the SMP of 90% BMA + 10% BPA with *T*_*g*_ = 56 °C, *R*_*f*_ is decreased to nearly zero when *T*_*D*_ is 25 °C where the polymer chain mobility is significantly reduced and the unrelaxed nonequilibrium stresses are stored as elastic energy.

Figure 3d indicates that the free shape recovery is a function of recovery temperature (Details about how to achieve the free recovery curve are presented in S2.1). We define the shape recovery ratio as *R*_*r*_ = 1−*e*(*t*)/(*e*_max_−Δ*e*). We use the recovery time *t*_0.95_ that corresponds to a 95% shape recovery ratio to measure the shape recovery rate at different recovery temperatures *T*_*R*_^24^. Within the lab scale experiment time (an hour), the SMP samples were able to realize the 95% shape recovery only at the recovery temperature *T*_*R*_ more than 10 °C higher than the SMP’s *T*_*g*_ (the measured *t*_0.95_ are listed in Table S5). The multi-branch model is also used to simulate the free recovery at different recovery temperatures *T*_*R*_ and predict the recovery time *t*_0.95_ at different *T*_*R*_ s (See Supplementary Material S2.4). Overall, *t*_0.95_ increases exponentially with a decreasing *T*_*R*_, and for different SMPs, at the same *T*_*R*_, the one with a higher *T*_*g*_ requires a longer recovery time.

### Three dimensional printed structures with a single SMP

**Figure 4:**
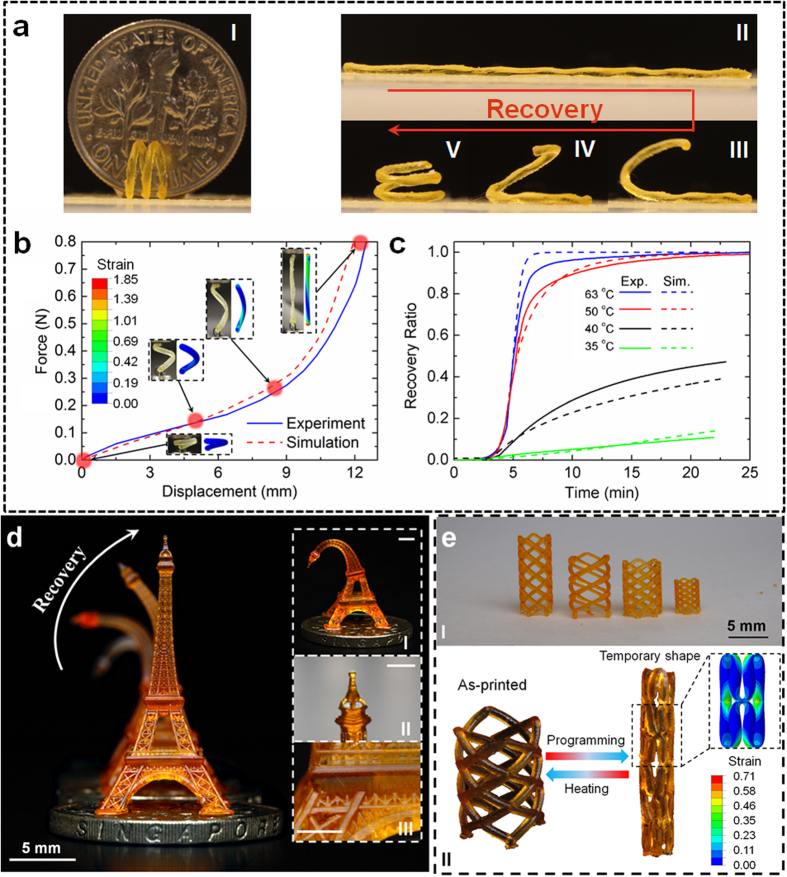
3D printed shape memory structures with single material. (**a**) A 3D printed shape memory spring (I) was programmed to a straight strand temporary configuration (II), and then recovered to its original shape upon heating (III–V). (**b**) Experimental characterization and FE simulation were performed to investigate the nonlinear deformation. (**c**) Experiments and simulations of the free recovery at different temperatures. (**d**) 3D printed SM Eiffel tower. (**e**) 3D printed SM stents.

Figure 4 shows the ability of our additive manufacturing system to create complex 3D structures that exhibit nonlinear large deformation SM behavior. As shown in Fig. 4aI, a spring was fabricated using a SMP with *T*_*g*_ = 43 °C (80% B + 20% P750). We demonstrated the SM effect of the spring by stretching it to a straight strand at 60 °C. The straight strand configuration was fixed (Fig. 4aII) after removing the external load at 20 °C. It recovered the original spring shape (Fig. 4aII–V) after heating back to 60 °C. The complicated nonlinear large deformation SM behavior of the spring was investigated by following the typical SM cycle for a spring with a representative segment (See Supplementary Material S3.3). Figure 4b shows the force-displacement relation when the spring was stretched at 60 °C. The spring becomes extremely stiff as it approaches to its fully unfolded state. The finite element (FE) simulations present the deformation contours in the progress of stretching the spring. Regardless of the maximum deformation on the two ends, the highest principle engineering strain on the main body of the spring was in the range from 70–100% which is about two to three times higher than the failure strain of previously reported SMPs used for 4D printing^2^, indicating the enhanced mechanical performance which is a necessity for active structures. In “4D printing” where the fourth dimension is “time”, a key desire is to control of the actuation rate. For the printed shape memory structures, the actuation rate can be controlled by the recovery temperature^24^,^49^. Figure 4c shows the recovery ratio of the stretched spring at different recovery temperatures. Here, the recovery ratio of the SM spring is defined as 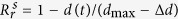, where *d*(*t*) is the end-to-end displacement during heating, *d*_max_ is the maximum displacement before unloading at 20 °C, and Δ*d* is the bounce back displacement after unloading. As seen in Fig. 4c, 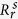 is highly dependent on the recovery temperature. At 60 °C, the spring was fully recovered into the initial shape within 3 mins. The recovery rate was significantly slower at 35 °C where only about 10% recovery took place after 20 mins holding. The SM behavior including the free recovery at different temperatures of the 3D printed spring can be simulated by implementing the multi-branch model into FE software ABAQUS (Simulia, Providence, RI, USA). In Fig. 4c, the FE simulation reproduced the free recovery behavior at different recovery temperatures, indicating that the multi-branch model can be used to design complex 4D printed structures that are made of SMPs and exhibit complex nonlinear large deformation thermomechanical behaviors. Details about FE simulation of SM behavior of this printed spring are described in Supplementary Material S3.3 and Supplementary Movies 1a–d.

Figure 4d shows a more refined and complex 3D printed structure Eiffel Tower standing on a Singapore dollar. It was printed with the SMP made of 80% B + 20% P750 too. Following the SM cycle, a temporary bent shape (Fig. 4dI) was achieved by bending the Eiffel tower at 60 °C and removing the external load after cooling to 25 °C. After heating back to 60 °C, the bent Eiffel tower gradually recovered its original straight shape (Fig. 4d, Supplementary Movie 2). Figure 4e demonstrates one of the most notable applications of SMPs — cardiovascular stent. Although there have been various efforts directed at fabrication^12^,^32^, material and structural characterization^12^,^61^,^62^ and simulations^32^,^63^,^64^,^65^, the design of the stents has been limited primarily by fabrication methods because traditional manufacturing approaches are usually complex, consisting of multiple time-consuming steps, to achieve the geometric complexity and resolution necessary for stents^12^,^32^. Our additive manufacturing system offers the ability to fabricate high resolution 3D shape memory structures with hardly any restriction of geometric complexity. Figure 4eI shows an array of stents printed in one batch with different geometric parameters including the height and the diameter of a stent, the number of joints, the diameter of ligaments and the angle between ligaments. In Fig. 4eII, a 3D printed stent was programmed into the temporary shape with a smaller diameter for minimally invasive surgery. After heating, the stent was recovered into the original shape with a larger diameter used to expand a narrowed artery. The finite element (FE) simulation shown in Fig. 4eII gives an insight into the local large deformation that occurs in the temporary shape, and renders existing additive manufacturing systems and materials infeasible. The simulations provide a guide for the material selection based on the understanding of the thermomechanical properties from Fig. 2.

### Three dimensional printed structures with multiple SMPs

**Figure 5:**
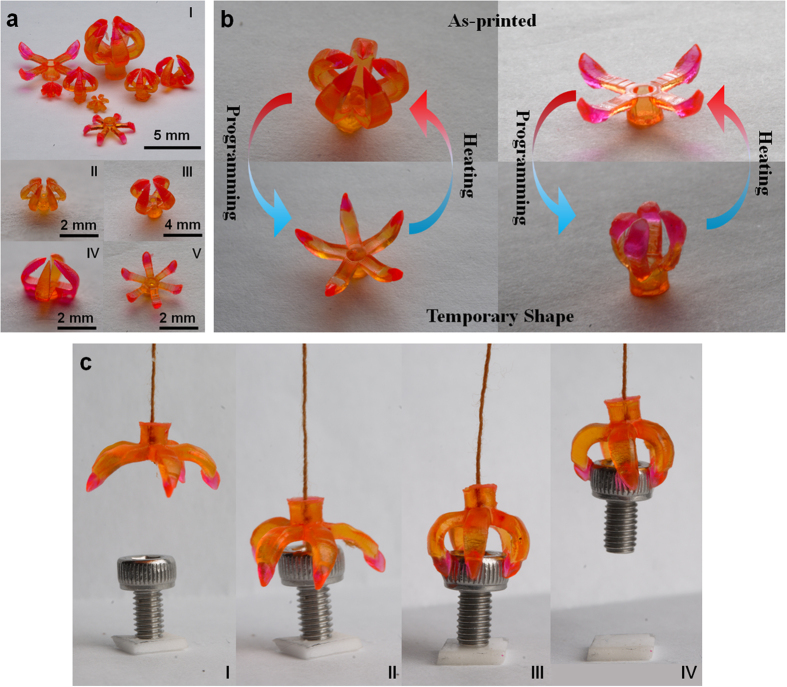
3D printed multimaterial grippers. (**a**) Multimaterial grippers were fabricated with different designs. (**b**) The demonstration of the transition between as printed shape and temporary shape of multimaterial grippers. (**c**) The snapshots of the process of grabbing an object.

Figure 5 demonstrates the printing of a 3D printed structure with multiple SMPs - multimaterial grippers that have the potential to function as microgrippers^13^ that can grab objects, or drug delivery devices^33^,^66^ that can release objects. Figure 5aI shows a number of multimaterial grippers with different designs including different sizes and numbers of digits (comparing Fig. 5aII and III), multiple materials placing at different positions (Fig. 5aIII and IV), and different mechanisms of the grippers to enable different functionalities (the closed grippers in Fig. 5aIII for grabbing objects and the open gripper in Fig. 5bV for releasing objects). In Fig. 5b, an as-printed closed (open) gripper was opened (closed) after programming and the functionality of grabbing (releasing) objects was triggered upon heating. Figure 5c shows time-lapsed images of a gripper grabbing an object (Supplementary Movies 3).

Compared to contemporary manufacturing approaches^13^,^33^,^66^ that essentially realized the gripper deformation of folding or unfolding by creating strain mismatches between layers of a thin multilayer hinge with thickness from a few microns to a few hundred nanometers which is about 1000 times smaller than the size of the entire structure^13^,^33^,^66^, our approach is simple and straightforward enabling stiffer grippers with thick joints made of SMPs. Additionally, the capability of multimaterial fabrication enables us to print the tips of the grippers with the materials different from the SMPs constructing the joints, and to design the stiffness of the tips based on that of the object to realize a safe contact. Details about material selections of the 3D printed grippers are described in Supplementary Material S4.1.

**Figure 6:**
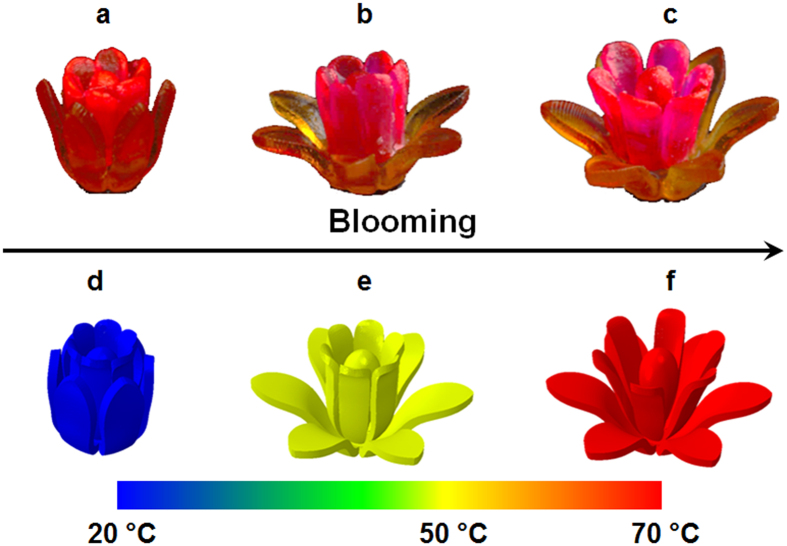
The sequential recovery of a multimaterial flower. The multimaterial flower in the original shape (**c**) was first programmed into the temporary bud state at 20 °C (**a**). The outer petals opened first after heating to 50 °C (**b**) and then, the flower fully bloomed at 70 °C (**c**). (**d**)–(**f**) represent the FE simulations of the corresponding flower blooming process.

Finally, by controlling the dynamic properties of the different SMPs as investigated in Fig. 3d, we are able to design the time dependent sequential shape recovery^4^,^67^ of a structure fabricated with multiple SMPs. In Fig. 6, we demonstrate sequential shape recovery by printing a multimaterial flower whose inner and outer petals have different *T*_*g*_ s (inners petal made of 90% B + 10% BPA with *T*_*g*_ = 56 °C and outer petals made of 80% B + 20% P750 with *T*_*g*_ = 43 °C). We first closed all the petals at 70 °C, and then decreased the temperature to 20 °C. After removal of the external constraint, the flower was fixed at the temporary bud state (Fig. 6a) where both the inner and outer petals stayed closed. The sequential recovery was triggered by raising the temperature first to 50 °C at which only the outer petals opened. The inner petals with *T*_*g*_ of 56 °C opened later after temperature was raised to 70 °C, completing the full shape recovery of the flower to its original blooming state (Fig. 6c). In Fig. 6d–f, a FE simulation (details can be founded in Supplementary Material S4.2) predicts this flower blooming process indicating that the multi-branch model can be used to design complex 4D printed structures that are made of multiple SMPs and exhibit sequential shape.

## Methods

### Development of multimaterial fabrication system

To develop a high resolution multimaterial system based on P*μ*SL, a CEL5500 LED light engine purchased from Digital Light Innovation (Austin, Taxes, USA) was used to work as the digital micro-display, a translation stage (LTS300) with 0.1 *μ*m minimum achievable incremental movement and 2 *μ*m backlash purchased from Thorlabs (Newton, New Jersey, USA) was used to work as the elevator, a stepper motor purchased from SparkFun Electornics (Niwot, Colorado, USA) controlled by Arduino UNO board works as a shaft to build the automated material exchange system. A custom LabView code was developed to control all the electronic components and automate the printing process.

### Material synthesis

All the chemicals including the methacrylate based monomers and crosslinkers, photo initiator, and photo absorbers were purchased from Sigma Aldrich (St. Louis, MO, USA) and used as received. Phenylbis (2, 4, 6-trimethylbenzoyl) phosphine oxide works as photo initiator mixed into the methacrylate based polymer resolution at the concentration of 5% by weight. Sudan I and Rhodamine B works as photo absorber fixed at concentration of 0.05% and 1% by weight, respectively.

### Printing and post-processing

The designed 3D structures were first sliced into layers with a prescribed layer thickness (most structures here were sliced with 50 *μ*m per layer). The custom LabVIEW with printing parameters which specify layer thickness, light intensity, exposure time, sends the sliced 2D images in order to digital micro display and controls the light irradiation of the digital micro displace, and translational stage motion. Once the 3D structures were printed, they were rinsed by the ethanol solution to remove the extra unreacted polymer solution. After that, the 3D structures were placed into a UV oven (UVP, Ultraviolet Crosslinkers, Upland, CA, USA) for 10 min post-curing.

## Additional Information

**How to cite this article**: Ge, Q. *et al.* Multimaterial 4D Printing with Tailorable Shape Memory Polymers. *Sci. Rep.* **6**, 31110; doi: 10.1038/srep31110 (2016).

## Supplementary Material

Supplementary Information

Supplementary Movie S1

Supplementary Movie S2

Supplementary Movie S3

Supplementary Movie S4

Supplementary Movie S5

Supplementary Movie S6
