# Supplementary material for: Multimaterial 4D Printing with Tailorable Shape Memory Polymers
Source: Sci Rep. 2016 Aug 8;6:31110. doi: 10.1038/srep31110 (PMC4976324; doi:10.1038/srep31110)
Supplement: Supplementary Information [file srep31110-s1.doc]

**Supplementary Materials**

**Multimaterial 4D Printing with Tailorable Shape Memory Polymers**

Qi Ge ^1, 2 *^, Amir Hosein Sakhaei ^1^, Howon Lee ^2^, Conner K Dunn ^3^, Nicholas X. Fang ^2^ *,

and Martin L. Dunn ^1 *^

^1^ Digital Manufacturing and Design Center, Singapore University of Technology and Design, Singapore

^2^ Department of Mechanical Engineering, Massachusetts Institute of Technology, Cambridge, MA, 02139, USA.

^3^ The George W. Woodruff School of Mechanical Engineering, Georgia Institute of Technology, Atlanta, GA, 30332, USA

* Contact information of corresponding authors:

Qi Ge (email: [ge_qi@sutd.edu.sg](mailto:ge_qi@sutd.edu.sg)), Nicholas X. Fang (email: [nicfang@mit.edu.sg](mailto:nicfang@mit.edu.sg)) and Martin L. Dunn (email: [martin_dunn@sutd.edu.sg](mailto:martin_dunn@sutd.edu.sg))

**S1 Experimental Characterization**

**S1.1 Dynamic mechanical analysis**

We used a dynamic mechanical analysis (DMA) tester (Q800 DMA, TA Instruments) to characterize the thermomechanical properties of all the printed shape memory polymer (SMP) samples and structures. In the DMA tests, a 3D printed samples with the dimension 15 mm × 4 mm × 1mm was stretched by a dynamic tensile load at 1 Hz. The temperature was first equilibrated at a high one where sample was at its rubbery state for 10 mins, and then decreased to a low one where the sample was at its glassy state at a cooling rate of 2 °C/min. Figure 1Sa, b, c and d plot the DMA results including storage modulus along with Tan*δ* *vs* Temperature for the copolymer networks consisting of BMA+P550, BMA+P750, BMA+BPA, and BMA+DEG, respectively. The glass transition temperatures ($T_{g}$) were denoted at the peaks of Tan*δ*.


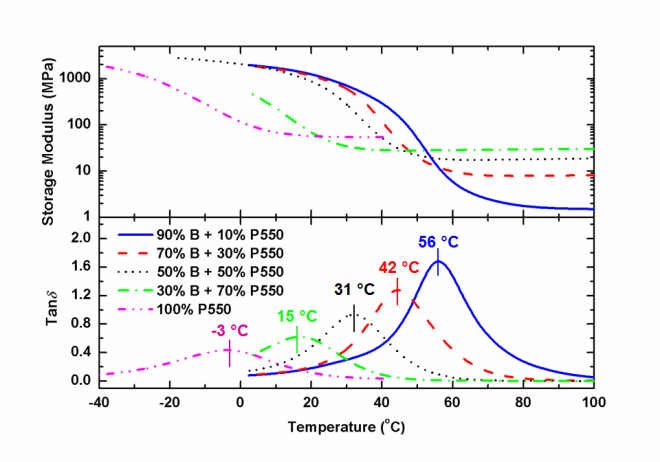

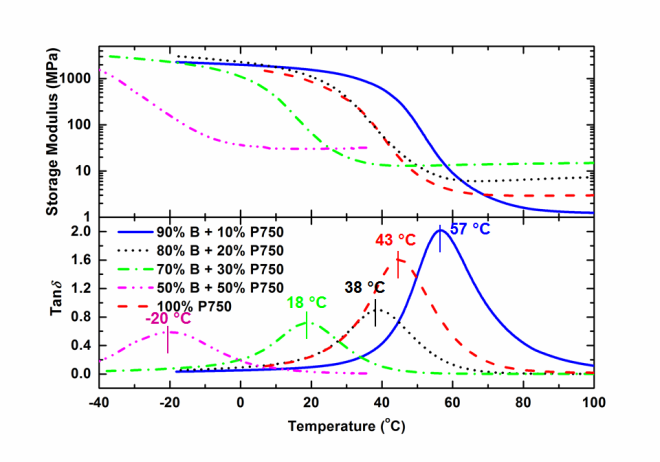


**a** **b**


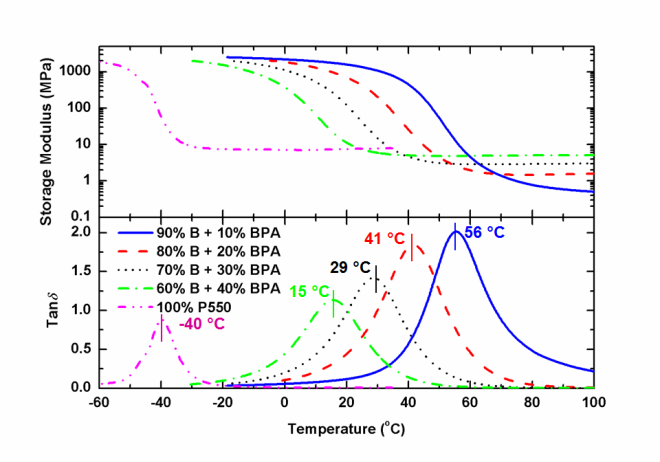

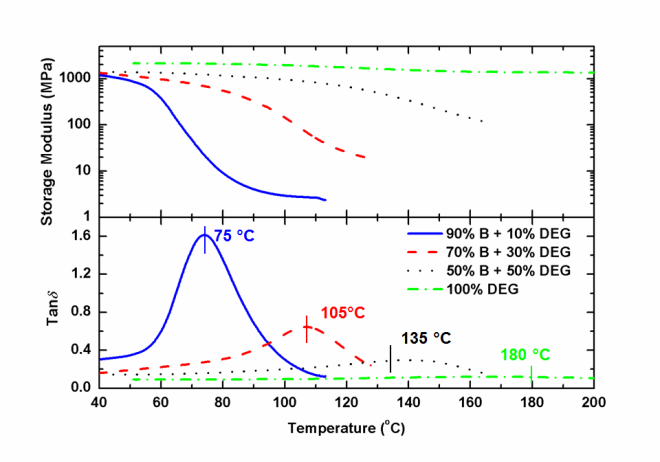


**c** **d**

**Figure S1|** **DMA tests of SMP samples**. **a.** BMA+PEGDMA550 networks. **b.** BMA+PEGDMA750 networks. **c.** BMA-BPA networks. **d.** BMA+DEGDMA networks.

**S1.2 Uniaxial tensile tests**

In the uniaxial tensile tests, the SMP samples made of different constituents and compositions were stretched at a loading rate of 0.1%/s at temperatures 30 °C higher that the glass transition temperature of each sample where the SMPs were at the rubbery state and purely elastic. Each SMP sample was tested three times and Figure S2 presents the one representative stress-strain behavior for these samples. The rubbery modulus for each sample was measured at 1% of the stress-strain curve. Tables S1 shows the mean values of rubbery modulus and failure strain for all the tested samples.


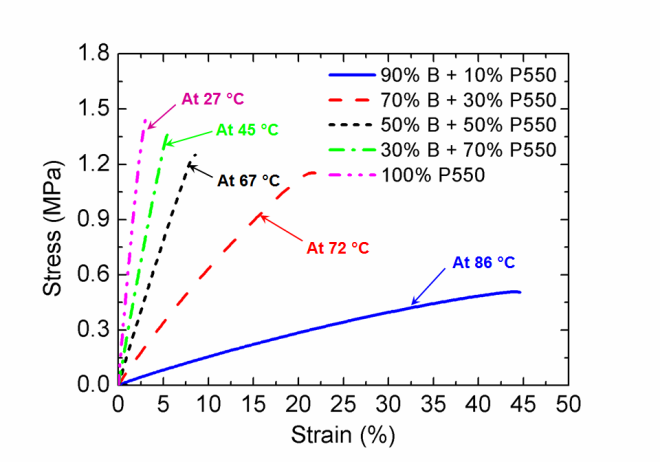

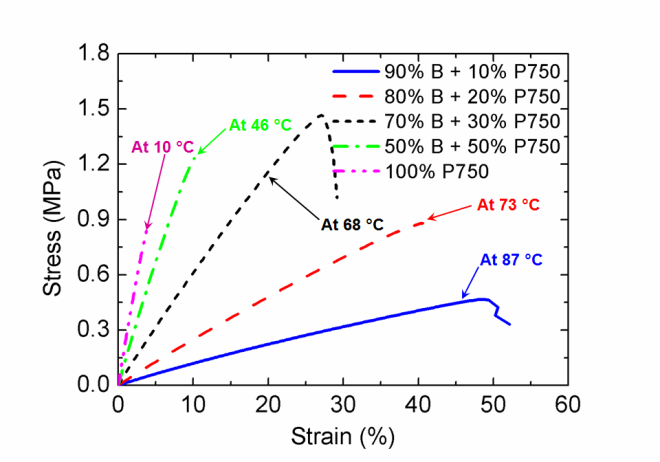


**a** **b**


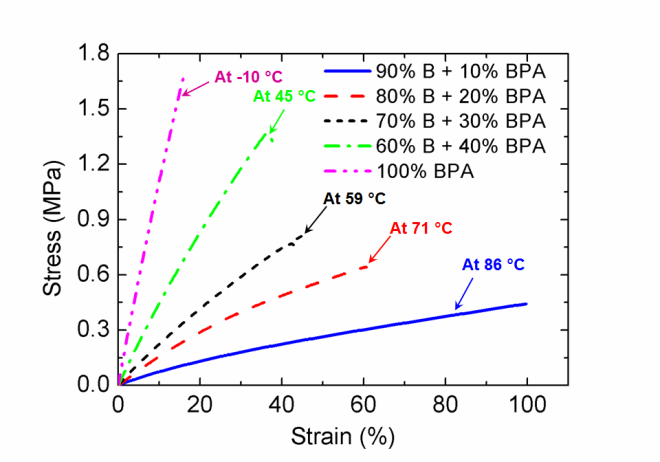

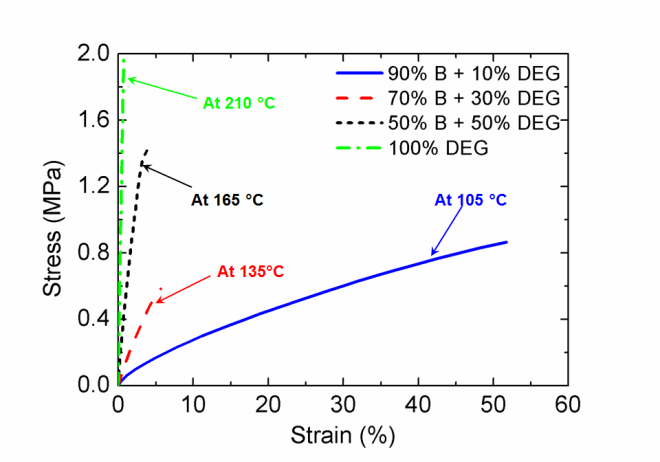


**c** **d**

**Figure S2| Uniaxial tensile tests of SMP samples.** **a.** BMA+PEGDMA550 networks. **b.** BMA+PEGDMA750 networks. **c.** BMA-BPA networks. **d.** BMA+DEGDMA networks.

**Table S1| Mean values of rubbery modulus and failure strain for all the tested SMP samples.**

| SMP Samples | $E_{r}$ (MPa) | $\varepsilon_{f}$ (%) |
| --- | --- | --- |
| 90% B + 10% P550 | 1.92±0.05 | 47.1±5.8 |
| 70% B + 30% P550 | 7.59±0.16 | 22.2±1.67 |
| 50% B + 50% P550 | 17.49±1.13 | 8.13±0.57 |
| 30% B + 70% P550 | 27.48±0.47 | 4.93±0.67 |
| 100% P550 | 52.2±3.55 | 3.1±0.361 |
| 90% B + 10% P750 | 1.31±0.04 | 48.17±3.61 |
| 70% B + 30% P750 | 2.85±0.1 | 36.73±1.43 |
| 50% B + 50% P750 | 7.13±0.1 | 27.63±2.87 |
| 30% B + 70% P750 | 14.44±0.39 | 12.35±1.08 |
| 100% P550 | 27.67±2.5 | 4.63±0.71 |
| 90% B + 10% BPA | 0.92±0.05 | 108.5±12.7 |
| 80% B + 20% BPA | 1.56±0.21 | 55.1±6.15 |
| 70% B + 30% BPA | 2.72±0.25 | 48.7 ±4.06 |
| 60% B + 40% BPA | 4.99±0.22 | 39.07±3.45 |
| 100% BPA | 10.93±0.6 | 15.47±3.11 |
| 90% B + 10% DEG | 5.18±0.35 | 44.2±6.56 |
| 70% B + 30% DEG | 20.67±2.36 | 8.7±0.25 |
| 50% B + 30% DEG | 79.45±0.75 | 4.17±0.35 |
| 100% DEG | 315.9±29.6 | 1.27±0.21 |

**S1.3 Conversion between mass fraction and molar fraction**

The conversion between mass fraction and molar fraction of crosslinkers is given in Table S2. Here, the molecular weights of BMA, PEGDMA 550, PEGDMA 750, BPA, DEGDMA are 176.1 g/mol, 550 g/mol, 750 g/mol, 1700 g/mol, 242.3 g/mol, respectively.

**Table S2| The conversion between mass fraction and molar of crosslinkers.**

| Crosslinker (wt %) | Crosslinker (mol %) | | | |
| --- | --- | --- | --- | --- |
|  | PEGDMA 550 | PEGDMA 750 | BPA | DEGDMA |
| 0 | 0 | 0 | 0 | 0 |
| 10 | 3.43536 | 2.542556 | 1.137884 | 7.471996 |
| 20 | 7.411304 | 5.544536 | 2.524333 | 15.37588 |
| 30 | 12.06633 | 9.142827 | 4.250782 | 23.75022 |
| 40 | 17.59065 | 13.5347 | 6.459778 | 32.63831 |
| 50 | 24.25286 | 19.01523 | 9.386493 | 42.08891 |
| 60 | 32.44488 | 26.04644 | 13.44857 | 52.15717 |
| 70 | 42.76199 | 35.39495 | 19.46563 | 62.9057 |
| 80 | 56.15434 | 48.43234 | 29.29629 | 74.40583 |
| 90 | 74.23767 | 67.87871 | 48.24804 | 86.73927 |
| 100 | 100 | 100 | 100 | 100 |

**S1.4 Temperature effect on failure strain**

We uniaxially stretched two SMP samples (90% BMA+10% BPA and 80% BMA+20% P750) under a loading rate of 0.1 %/s at different temperatures to investigate the temperature effect on the failure strain. As seen in Figure S3a, for the SMP sample made of 90% BMA and 10% BPA, the stress-strain curves from the tests at 60 °C, 70 °C 80 °C, and 86 °C exhibit a nearly linear behavior and overlaps with the same modulus indicating that above 60 °C, the SMP is at the rubbery state. However, within this temperature range, with the increase of the testing temperature, the failure strain drops dramatically from ~200% at 60 °C to ~90 % at 86 °C. At the testing temperatures between 50 °C to 40 °C where the SMP enters the temperature zoom of the transition from the rubbery state to the glassy state, the stress-strain curves of the SMP exhibit the nonlinear viscoelastic behavior, and the failure strain increases drastically and researches the maximum of ~340 % at 45 °C. While the SMP enters the glass state where the temperature is lower than 30 °C, it becomes extremely brittle and the failure strain quickly drops to less than 100 %. In Figure S3c, the stress-strain curves of the SMP made of 80% BMA and 20% P750 follow the same trend by changing the temperature from 70 °C to 0 °C. As seen in Figure S3b and d comparing the failure strain with the loss modulus from DMA tests, we found that the failure strain reaches the maximum at the temperature close to the peak of the loss modulus indicating that the highest energy dissipation leads to the highest stretchability.


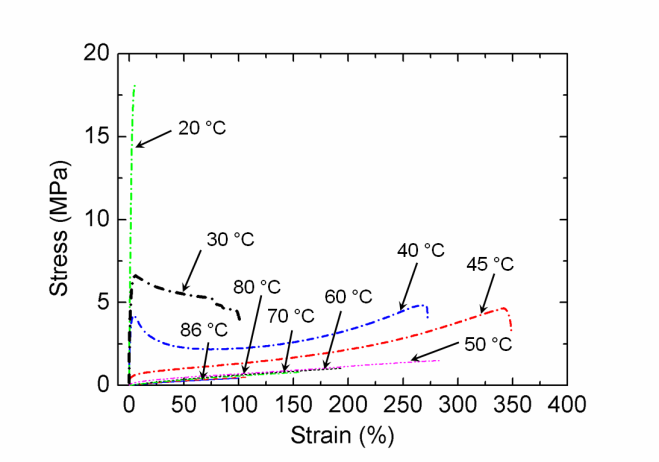

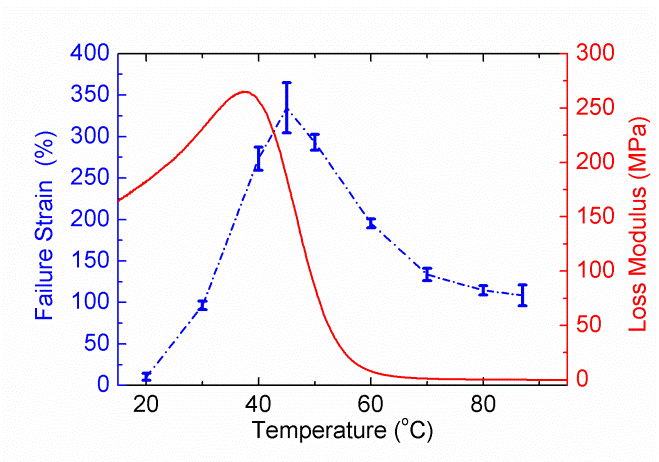


**a** **b**


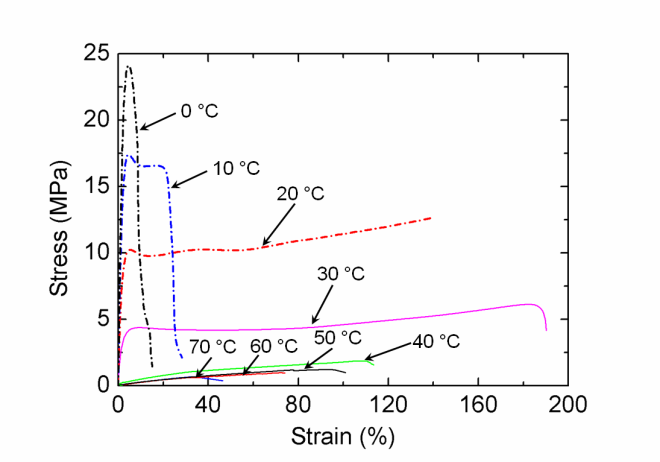

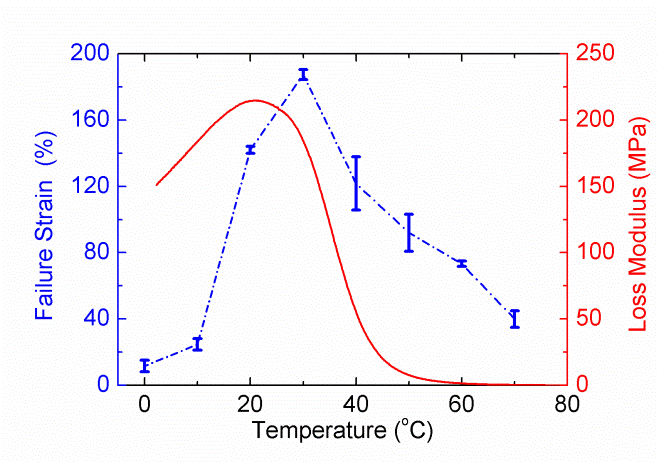


**c** **d**

**Figure S3| Temperature effect on failure strain.** **a** and **c** are the stress-strain curves achieved from unaxial tensile tests at different temperature of the SMP samples made of 90% BMA+10% BPA, and 80% BMA+20% P750, respectively. **b** and **d** compare the failure strains at different temperature to loss moduli achieved from DMA tests.

**S1.4 Effect of chemical composition on the kinetics of photopolymerization**

Experiments were conducted to study the effect of chemical composition on the kinetics of photo polymerization. The patterned UV light with energy 333 mJ/cm^2^ was exposed to polymer solutions that were deposited between two glass slides where two spacers with thickness of 50 *µ*m were placed between them. The UV light was turned off until the solidified pattern was visible and the time of UV light exposure was recorded. Figure S4a shows the light energy required to cure a 50 *µ*m thick pattern of SMP consisting of BMA and PEGDMA (550 and 750) with different crosslinker concentration. The normalized light energy was achieved by dividing actual light energy by the light energy for the SMP consisting of 90% BMA and 10% PEGDMA 750. The effect of the concentration of photo initiator on the kinetics of photo polymerization was investigated. The exposure time was recorded to cure a 50 *µ*m layer made of 70% BMA+ 30% P550 with the concentration of photoinitiator 0.5%, 2%, and 5% in terms of the total weight of the polymer solution. In Figure S4b, the exposure energy is significantly reduced when the concentration of photoinitiator is increased. In addition, we also compared the kinetics of photo polymerization between (meth)acrylate based polymer and acrylate based polymer. Figure S4c shows that to cure a 50 *µ*m (meth)acrylate layer made of 70% BMA+ 30% P550 requires the exposure energy as about 100 times high as that to cure the same thick layer made of poly(ethylene glycol) diacrylate with molecular weight 575 g/mol (PEGDA 575) indicating that the kinetics of photo-curing (meth)acrylate based polymer is much slower than that of acrylate based polymer.


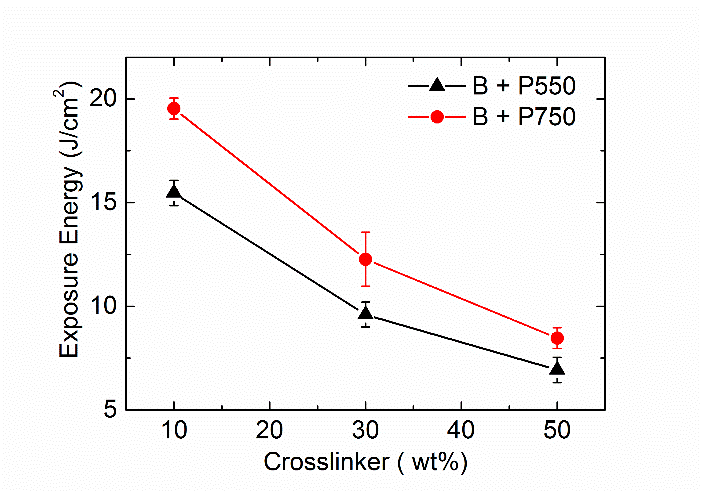

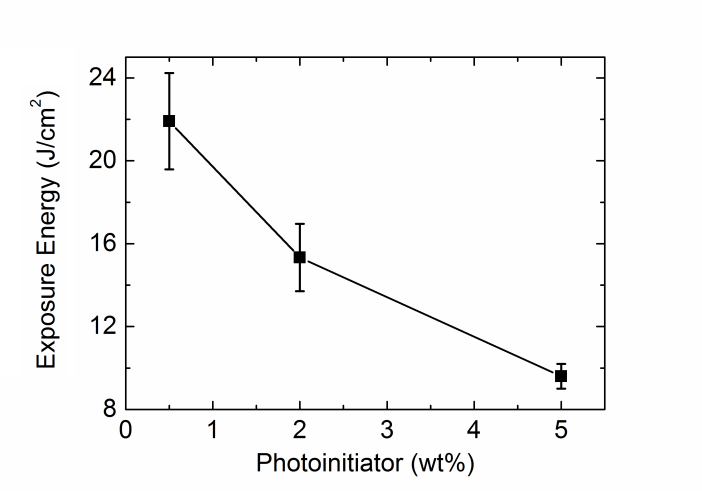


**a** **b**


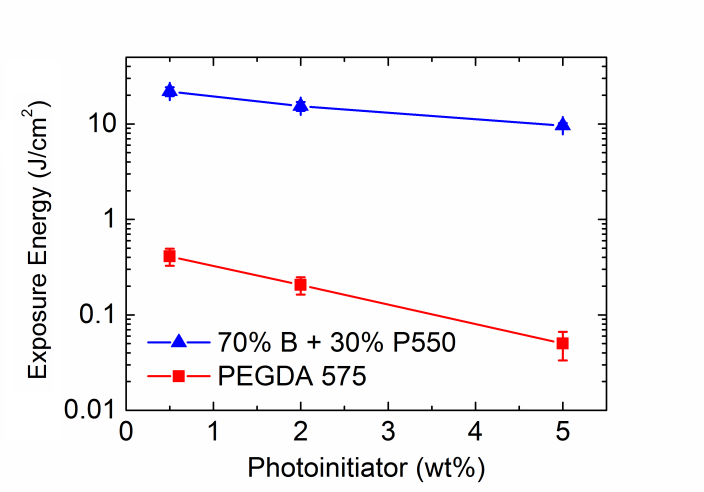


**c**

**Figure S4|** **Effect of chemical composition on the kinetics of photopolymerization. a.** The exposure energy for curing a 50 *µ*m layer varies with the concentration of crosslinker and the molecular weight of crosslinker. **b.** The exposure energy for curing a 50 *µ*m layer varies with the concentration of photoinitiator. **c.** Comparison of exposure energy for curing a 50 *µ*m layer made of 70% BMA+ 30% P550 and PEGDA 575.

**S1.5 Investigation of interface bonding between two SMPs**

The investigation of the interface bonding between two SMPs has been performed by uniaxially stretching a printed strip sample where the two components (Component A: 50% B+50% P550 with $T_{g}$ = 31 °C and Component B: 90% B+10% BPA $T_{g}$ = 56 °C) with volume fraction of 0.5 respectively arranged in series. In Figure S5a, the two peaks of Tan*δ* of the composite clearly indicate two separated thermal transitions, corresponding to the glass transitions of components A and B, respectively. As a result, the cascade of the three storage modulus plateaus of decreasing magnitude with increasing temperature was observed. The first high modulus plateau of the composite (~2 GPa) exists below $T_{g}$s of components A and B, where the both components are in the glassy state (Plateau I in Fig. S5a). The second moderate modulus plateau (~50 MPa) lies between $T_{g}$s of components A and B, where component A is in the rubbery state but component B is in the glass state (Plateau II in Fig. S5a). Above the $T_{g}$ of component B where the both components are in the glassy state, there is a low modulus plateau (~ 2MPa) (Plateau III, in Fig. S5a).

We uniaxially stretched the composite and the pure strip samples made of the two component materials respectively with a loading rate of 0.1%/s at 88 °C where the temperature is 30 °C higher than the $T_{g}$ of component B and all the tested samples are in the rubbery state. The Young’s modulus was measured where the samples were stretched by 1% and the failure strains were measured where the samples were fractured. During the uniaxial test, the composite breaks at Composite A that has a lower failure strain rather than at the interface, which indicates a strong interface bonding formed between the two components. The effective Young’s modulus of the composite also can be predicted by:

$\overline{E}=\frac{E_{A}E_{B}}{f_{A}E_{B}+f_{B}E_{A}}$, (S1)

where $E_{A}$ and $E_{B}$ are the measured Young’s modulus of components A and B, respectively. $f_{A}$ and $f_{B}$ are the volume fractions of components A and B, respectively. Here, $f_{A}$ = $f_{B}$ = 0.5. The effective failure strain of the composite also can be predicted by:

$\overline{\varepsilon}_{f}=\left\{\begin{matrix} \frac{f_{A}E_{B}+f_{B}E_{A}}{E_{B}}\cdot\varepsilon_{A},\text{ }\left(\text{breaks at A}\right), \\ \frac{f_{A}E_{B}+f_{B}E_{A}}{E_{A}}\cdot\varepsilon_{B}\text{, }\left(\text{breaks at B}\right), \end{matrix}\right.$ (S2)

where $\varepsilon_{A}$ and $\varepsilon_{B}$ are the measured failure strain of components A and B, respectively. If the composite breaks at composite A, the first equation will be used to calculate the effective failure strain. If the composite breaks at composite B, the second equation will be used.

Table S3 compares the measured and predicted effective Young’s modulus and failure strain of the composite. The good agreement between the measured and predicted values reveals that Components A and B form a strong covalently boned interface through which the composite transfers stress completely between the two components.


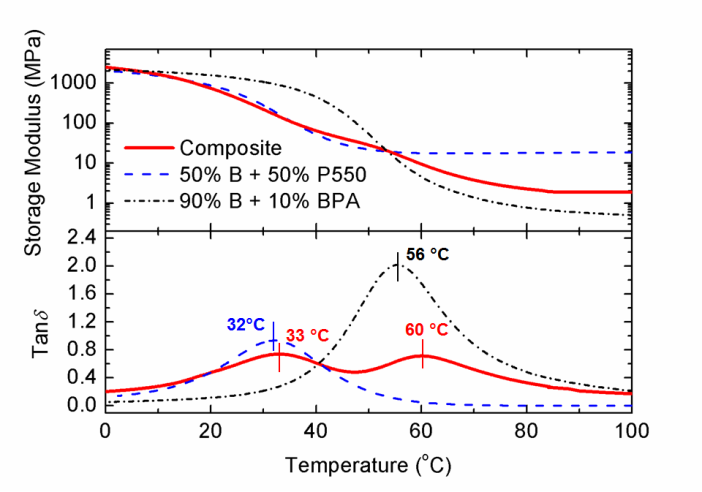

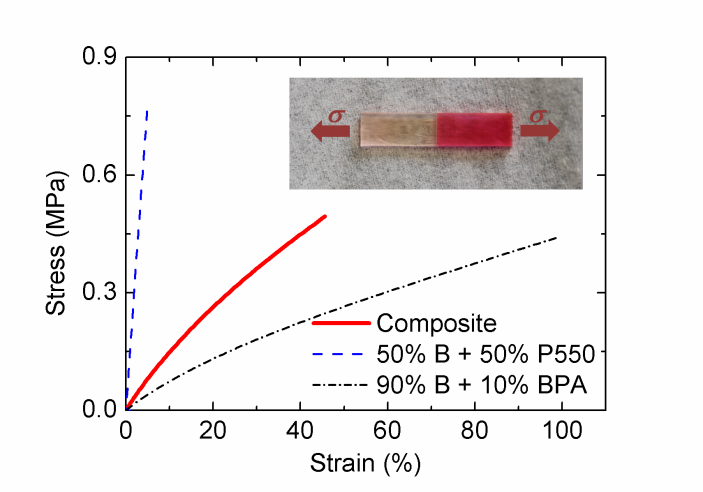


**a b**

**Figure S5| Thermomechanical tests to investigate the interface bonding between two SMPs. a.** DMA and **b**. uniaxial tensile tests of the composite and the components constructing the composite.

**Table S3| The measured and predicted Young’s modulus and failure strain.**

|  | Component A | Component B | Composite | |
| --- | --- | --- | --- | --- |
|  |  |  | Measured | Prediction |
| Modulus | 16.5 MPa | 0.92 MPa | 1.84 MPa | 1.74 MPa |
| Failure Strain | 5 % | 99 % | 46 % | 47.5 % |

**S2. Shape memory behavior**

**S2.1 Characterization of shape memory behavior**

The shape memory (SM) behavior of the SMP strips was investigated by following the typical shape memory cycling method shown in Figure S6a. Figure S6b presents the temperature conditions for testing the SMP samples made of 80% B+20% P750. A sample was first stretched to $e_{\max}$ (20%) with a constant loading rate (0.001 s^-1^) at a programming temperature $T_{D}$ (63 °C which is 20 °C higher than sample’s $T_{g}$), and then the temperature was decreased to a low one $T_{L}$ ($T_{L}$ = 25 °C) with a cooling rate 2.5 °C/min. Once $T_{L}$ reached, the sample was first held for 2 minutes, and then the tensile force was removed. In the free recovery step, the temperature was increased to a recovery temperature $T_{R}$ ($T_{R}$ = 35 °C, 40 °C, 50 °C, 60 °C, respectively) at the same rate of cooling and subsequently stabilized for another 20 min. Figure S6c shows the corresponding strain change during the shape memory cycle. The bouncing back strain $\Delta e$ was measured at Step III-unloading to calculate the shape fixity $R_{f}$. The sample was also programmed at lower $T_{D}$s ($T_{D}$ = 50 °C, 40 °C, and 35 °C, respectively), and the corresponding shape fixity was listed Table S4. The time-temperature dependent free recovery ratio is calculated by $R_{r}=1-e\left(t\right)/\left(e_{\max}-\Delta e\right)$ [^1^](#_ENREF_1) and shown in Figure S6d. Within the lab scale experiment time, the SMP sample was fully recovered only at the recovery temperature $T_{R}$ = 50 °C and 60 °C where we can extract the recovery times corresponding to 95% shape recovery ratio ($t_{0.95}$) that are listed in Table S5.

We also investigated the shape memory behavior the SMP samples made of 90 % B+10 % BPA ($T_{g}$ = 56 °C), and 60 % B+ 40 % P550 ($T_{g}$ = 31 °C), respectively. We studied the temperature effect on shape fixity by programming the sample made of 90 % B+10 % BPA at $T_{D}$ = 77 °C, 65 °C, 55 °C and 45 °C; programming the sample made of 60 % B + 40 % P550 at $T_{D}$ = 57 °C, 47 °C, 37 °C and 30 °C. We studied the temperature effect on free recovery by programming the sample made of 90 % B+10 % BPA at $T_{D}$ = 77 °C and recovering the sample at $T_{R}$ = 77 °C, 60 °C, and 50 °C; by programming the sample made of 60 % B+40 % P550 at $T_{D}$ = 57 °C and recovering the sample at $T_{R}$ = 57 °C, 50 °C, and 40 °C. The calculated $R_{f}$ and the measured readable $t_{0.95}$ are listed in Table S5.


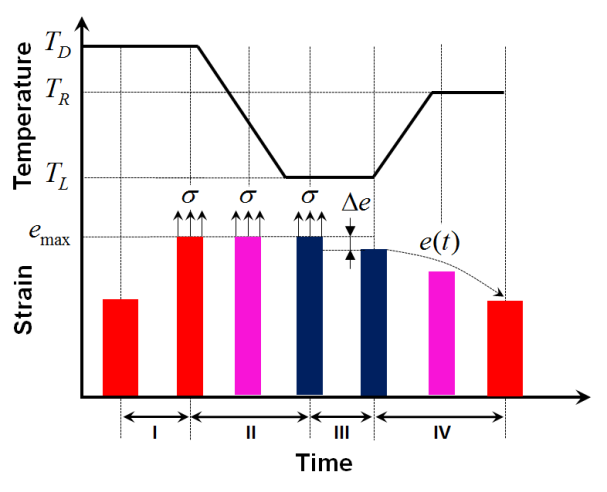

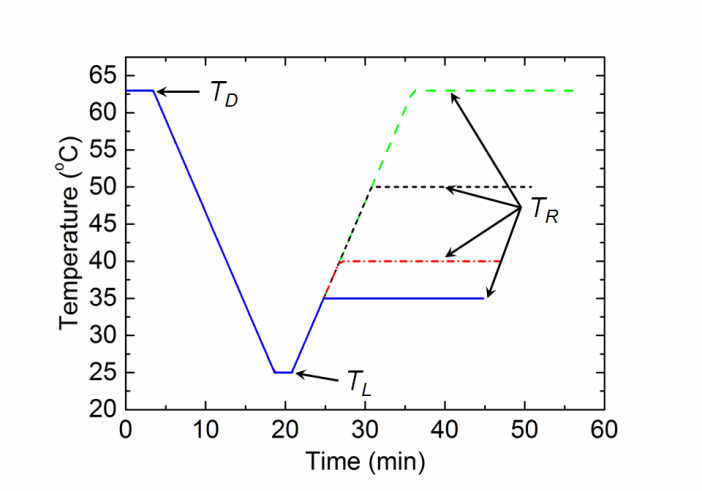


**a b**


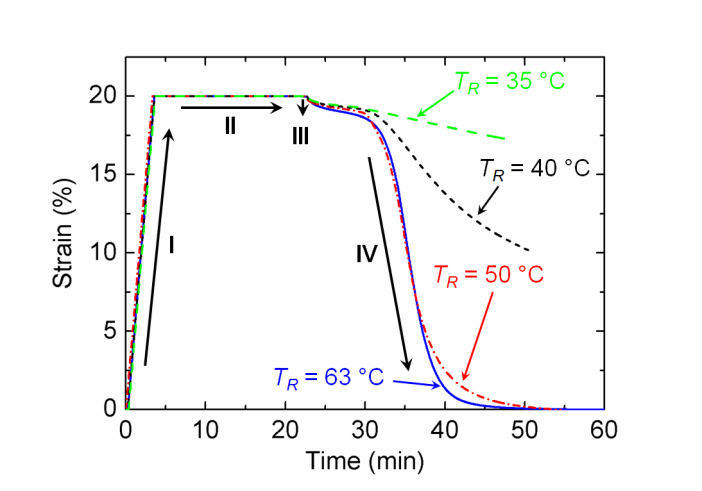

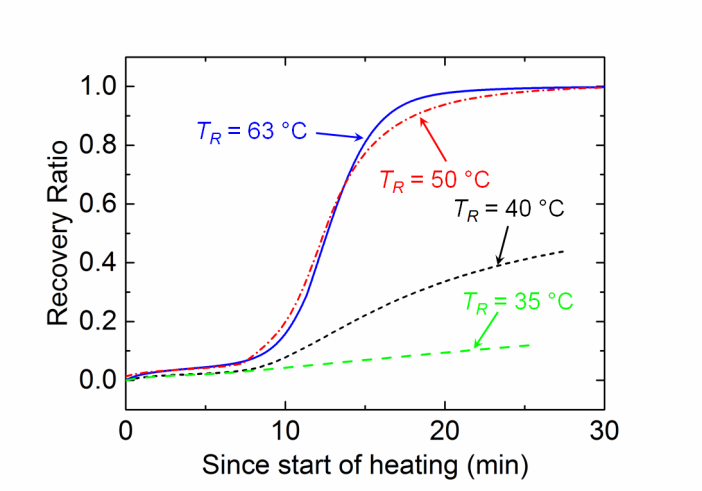


**c** **d**

**Figure S6| Experiments of SM behavior.** **a**. A typical SM cycle. **b**. The representative temperature conditions for testing the SMP sample made of 80% B+20% P750. **c**. The representative SMP strain-time curves for the SMP sample made of 80% BMA and 20% P750. **d**. The temperature dependent recovery ratio varies with time.

**Table S4| Shape fixity at different programming temperature $T_{D}$.**

|  | 90 % B + 10 % BPA | | | | 80 % B + 20 % P750 | | | | 60 % B + 40 % P550 | | | |
| --- | --- | --- | --- | --- | --- | --- | --- | --- | --- | --- | --- | --- |
| $T_{D}$ (°C) | 77 | 65 | 55 | 45 | 63 | 50 | 40 | 35 | 57 | 47 | 37 | 30 |
| $R_{f}$ (%) | 100 | 100 | 99 | 98 | 99 | 99 | 97 | 90 | 99 | 98 | 92 | 80 |

**Table S5| $t_{0.95}$ at different recovery temperature $T_{R}$.**

|  | 90 % B + 10 % BPA | | 80 % B + 20 % P750 | | 60 % B + 40 % P550 | |
| --- | --- | --- | --- | --- | --- | --- |
| $T_{R}$ (°C) | 77 | 60 | 63 | 50 | 57 | 50 |
| $t_{0.95}$ (min) | 19 | 21 | 17.9 | 21 | 12.4 | 13.7 |

**S2.2 Multi-branch model**

A multi-branch model [^1-3^](#_ENREF_1) has been used to simulate the shape memory behavior of the printed samples and structures. As shown in Figure S7, the total deformation gradient $F_{total}$ could be decomposed into the mechanical deformation gradient $F_{M}$ and the thermal deformation gradient $F_{T}$:

$F_{total}=F_{M}F_{T}$. (S3)

Here, the deformation gradient is defined by $F\text{=}\partial x/\partial X$, where $X$ is the initial position of a material point in the reference configuration and $x$ is the current position of that material point in the spatial configuration. The thermal expansion is assumed to be isotropic, i.e.,

$F_{T}=J_{T}I$, (S4)

where $I$ is the second order unit tensor and $J_{T}$ is the volume change due to thermal expansion, i.e.,:

$J_{T}=\frac{V\left(T\right)}{V_{0}}$, (S5)

where $V\left(T,t\right)$ is the instantaneous volume at temperature $T$, $V_{0}$ is the reference volume at the reference temperature $T_{0}$. The nonlinear volume change can also be simplified by a bilinear representation ,

$dV\left(T\right)=3\alpha_{r}dT,\text{ }\left(T>T_{g}\right)$ and $dV\left(T\right)=3\alpha_{g}dT,\text{ }\left(T\leq T_{g}\right)$, (S6)

where $\alpha_{r}$ and $\alpha_{g}$ for the coefficients of thermal expansion (CTE) of the rubbery state and the glassy state, respectively.

**
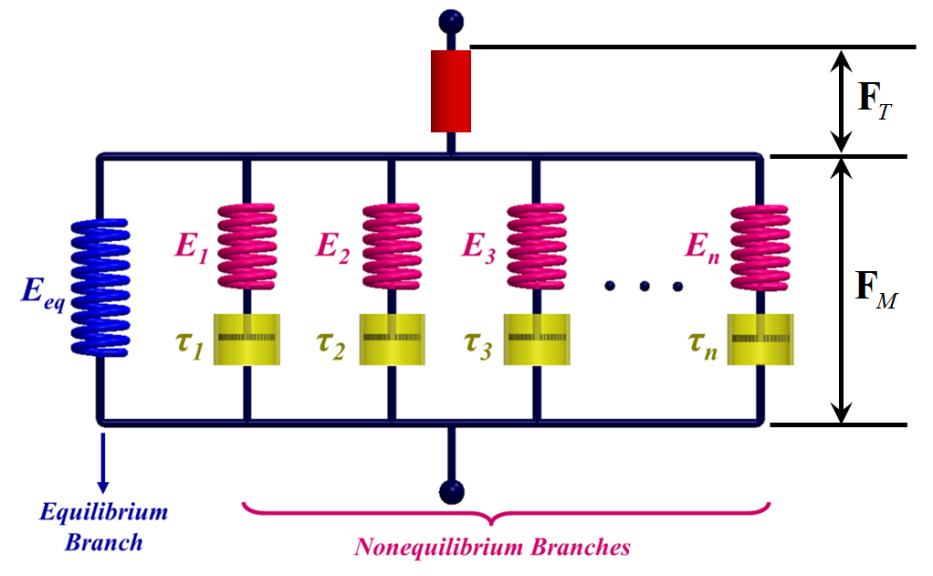
**

**Figure S7| 1D rheological representation of the multi-branch model.**

The mechanical deformation gradient, $F_{M}$, gives rise to the Cauchy stress $\sigma_{total}$that is applied to the material and can be expressed as:

$\sigma_{total}=\sigma_{eq}+\sum_{i=1}^{n} \sigma_{non}^{i}$, (S7)

where $\sigma_{eq}$ and $\sigma_{non}^{i}$ are Cauchy stresses in equilibrium branch and the *i*th nonequilibrium branch, respectively. Here, the neo-Hookean model is adopted to define $\sigma_{eq}$:

$\sigma_{eq}=\frac{Nk_{B}T}{J_{M}}\left(\bar{B}-\frac{1}{3}\text{tr}\bar{B}\right)+K_{eq}\left(J_{M}-1\right)I$, (S8)

where $N$ is the crosslinking density, $k_{B}$ is Boltzmann’s constant, $T$ is the absolute temperature. $Nk_{B}T$ is the temperature dependent shear modulus, which indicates entropic elasticity. $K_{eq}$ is the bulk modulus of the equilibrium branch, which is typically orders of magnitude larger than the shear modulus. $\bar{B}$ is the modified left Cauchy-Green tensor given by $\bar{B}=\bar{F}_{M}\left(\bar{F}_{M}\right)^{T}$, with $\bar{F}_{M}=\left(J_{M}\right)^{-1/3}F_{M}$ and $J_{M}=\det\left(F_{M}\right)$.

For the nonequilibrium behavior in the *i*th viscoelastic branches, the deformation gradient can be further decomposed into an elastic part and a viscous part:

$F_{M}=F_{e}^{i}F_{v}^{i}$, (S9)

where $F_{v}^{i}$ is a relaxed configuration obtained by elastically unloading by $F_{e}^{i}$. The Cauchy stress on *i*th nonequilibrium branch, $\sigma_{non}^{i}$, is given by:

$\sigma_{non}^{i}=\frac{1}{J_{e}^{i}}\left(L_{e}^{i}:\ln V_{e}^{i}\right)$, (S10)

where $J_{e}^{i}=\det\left(F_{e}^{i}\right)$, $V_{e}^{i}=F_{e}^{i}\left(R_{e}^{i}\right)^{T}$ and $R_{e}^{i}$ is the rotation tensor. $L_{e}^{i}$ is the fourth order isotropic. The viscous deformation gradient $F_{v}^{i}$ is time and temperature dependant, and related the temperature dependent relaxation time $\tau_{i}\left(T\right)$ which can be obtained from the reference time using a shifting factor $a_{T}$:

$\tau_{i}\left(T\right)=a_{T}\tau_{0i}$, (S11)

where $\tau_{0i}$ is the stress relaxation time at a reference temperature. It was found that depending on whether temperature is above, near, or below $T_{g}$, the shifting factor $a_{T}$ can be calculated by two different method [^5^](#_ENREF_5):

$a_{T}=\left\{\begin{matrix} 10^{-\text{ }\frac{C_{1}\left(T-T_{r}\right)}{C_{2}+\left(T-T_{r}\right)}} & T\geq T_{r} \\ e^{-\text{ }\frac{AF_{C}}{k}\left(\frac{1}{T}-\frac{1}{T_{r}}\right)} & T<T_{r} \end{matrix}\right.,$ (S12)

where $C_{1}$, $C_{2}$ and $A$ are material constants, $F_{c}$ is the configuration energy, $k$ is Boltzmann’s constant and $T_{r}$ is the reference temperature. Details about the finite deformation multi-branch model are described by Westbrook et al. [^3^](#_ENREF_3).

**S2.3 Parameter identification**

A one-dimensional analytical solution [^1^](#_ENREF_1) for this multi-branch model was used in paper to predict shape fixity and free recovery for SMP strip samples under different thermomechanical conditions. The parameters in the multi-branch model can be simply identified by a set of stress relaxation tests (Figure S8a). Here, we take the SMP sample made of 80% B+20% P750 example. A stress relaxation master curve at 31 °C in Figure S8b was constructed by shifting relaxation curves using shift factors ($a_{T}$) at different temperatures (Figure S8c). The master curve can be described by Maxwell elements in parallel and the stress relaxation modulus is:

$E(t)=E_{\infty}+\sum_{i=1}^{n} E_{non}^{i}\exp\left[-\left(\frac{t}{\tau_{m}^{R}}\right)\right]$ with $\tau_{i}^{R}=10^{i-1}\tau_{1}^{R}\text{, }\left(i=2,...,n\right)$. (S13)

In Eq. (S13), $E_{\infty}$ is the relaxation modulus at time $t$ = $\infty$ ($E_{\infty}$~ 2.5 MPa in Figure S8b); $\tau_{i}^{R}$ is the relaxation time for the *i-*th branch at the reference temperature (31 °C). We assume that the relaxation time of the *i-*th branch is a decade longer than the (*i-*1)-th branch. At time $t$ = 0, the relaxation modulus is $E\left(0\right)=E_{\infty}+\sum_{i=1}^{n} E_{non}^{i}$. Following the previously reported parameter fitting approach , Eq. (S13) can nicely fit the stress relaxation master curve at 31 °C (Figure S8c) with the fitted nonequilibrium modulus $E_{non}^{i}$ and stress relaxation time $\tau_{i}^{R}$ for the *i-*th branch. The parameters $C_{1}$, $C_{2}$, $AF_{c}/k$ in Eq. (S12) can be obtained by fitting the shift factor-temperature curve (Figure S8c). All values of parameters for SMPs made of 80% B+20% P750, 90% B+10% BPA, and 60% B+40% P550 are listed in Table S6.


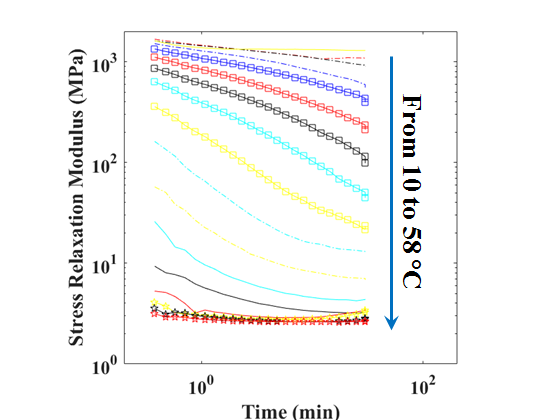

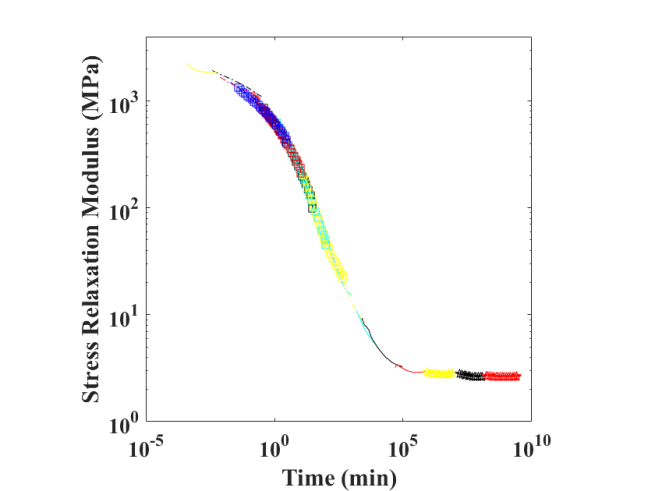


**a b**


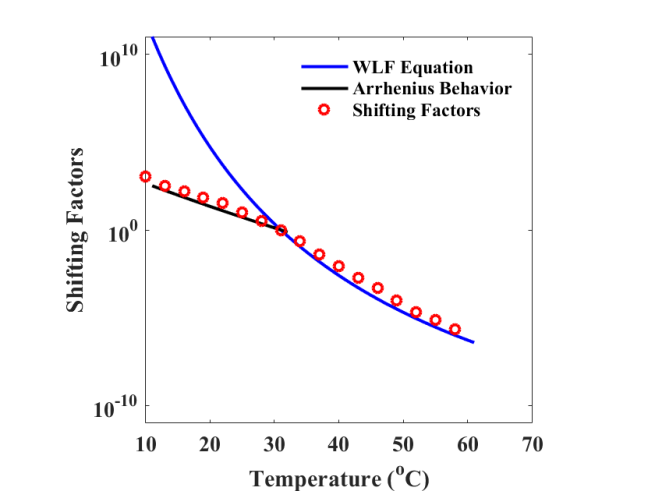

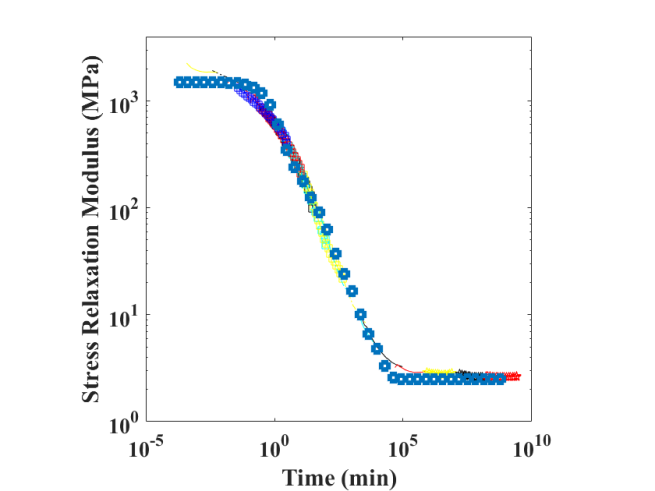


**c d**

**Figure S8| Model fitting for stress relaxation.** **a.** Stress relaxation tests for the SMP sample made of 80% B+20% P750 from 10 °C – 58 °C with of interval of 3 °C. **b.** The stress relaxation master curve at 31 °C. **c.** The shifting factors with temperature. **d.** Model fitting of the stress relaxation mater curve.

**Table S6| Parameters of the multi-branch model.**

| 80% B + 20% P750 | | | | | | | | | | | | | |
| --- | --- | --- | --- | --- | --- | --- | --- | --- | --- | --- | --- | --- | --- |
| $E_{\infty}$ | $E_{non}^{1}$ | $E_{non}^{i}$ | $\tau_{1}^{R}$ | $\tau_{i}^{R}$ | | $T_{R}$ | | $C_{1}$ | | $C_{2}$ | | $AF_{c}/k$ | |
| 2.5 MPa | 800 MPa | $E_{non}^{1}/2^{i-1}$,  ($i$ = 2, …, 5) | 60 s | $\tau_{1}^{R}/10^{i-1}$,  ($i$ = 2, …, 5) | | 31 °C | | 17.44 | | 51.6 °C | | 35000 K | |
| 90% B + 10% BPA | | | | | | | | | | | | | |
| $E_{\infty}$ | $E_{non}^{1}$ | $E_{non}^{i}$ | $\tau_{1}^{R}$ | | $\tau_{i}^{R}$ | | $T_{R}$ | | $C_{1}$ | | $C_{2}$ | | $AF_{c}/k$ |
| $\begin{matrix} T_{r}=42^{\circ}\text{C,} \\ C_{1}=13.44, \\ C_{1}=55.6, \\ \frac{AF_{c}}{k_{b}}=50000 \end{matrix}$2 MPa | 1200 MPa | $E_{non}^{1}/2^{i-1}$,  ($i$ = 2, …, 5) | 0.006 s | | $\tau_{1}^{R}/10^{i-1}$,  ($i$ = 2, …, 5) | | 42 °C | | 13.44 | | 55.6 °C | | 50000 K |
| 60% B + 40% P550 | | | | | | | | | | | | | |
| $E_{\infty}$ | $E_{non}^{1}$ | $E_{non}^{i}$ | $\tau_{1}^{R}$ | | $\tau_{i}^{R}$ | | $T_{R}$ | | $C_{1}$ | | $C_{2}$ | | $AF_{c}/k$ |
| $\begin{matrix} T_{r}=42^{\circ}\text{C,} \\ C_{1}=13.44, \\ C_{1}=55.6, \\ \frac{AF_{c}}{k_{b}}=50000 \end{matrix}$30 MPa | 2400 MPa | $E_{non}^{1}/2^{i-1}$,  ($i$ = 2, …, 5) | 0.6 s | | $\tau_{1}^{R}/10^{i-1}$,  ($i$ = 2, …, 5) | | 24 °C | | 16.44 | | 55.6 °C | | 35000 K |

$$\begin{matrix} T_{r}=24^{\circ}\text{C,} \\ C_{1}=16.44, \\ C_{1}=55.6, \\ \frac{AF_{c}}{k_{b}}=35000 \end{matrix}$$

**S2.4 Model prediction**

The one-dimensional multi-branch model with parameters listed in Table S6 can be used to predict the SM behavior of the SMP sample made of 80% B and 20% P750. In Figure S9, the model predictions show good agreement with experiments of SM behavior as well as free recovery. The model can be further used to predict the shape fixity under different programming temperature $T_{D}$s. Also, it can be used to predict the recovery times of 95% recovery at lower recovery temperature $T_{R}$ which takes longer time than lab time scale.


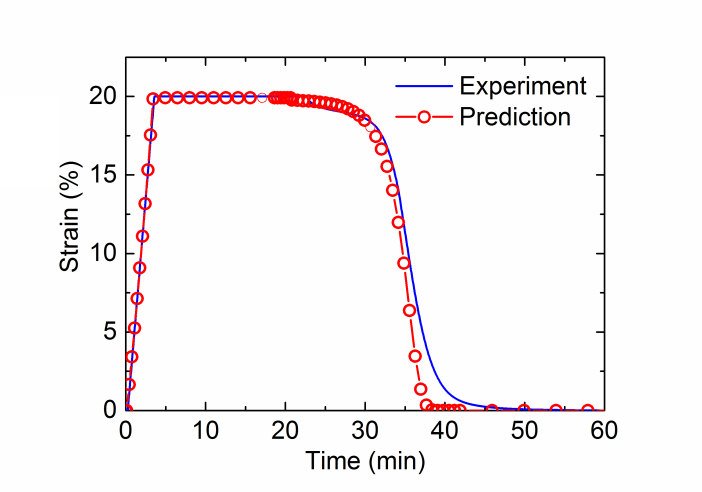

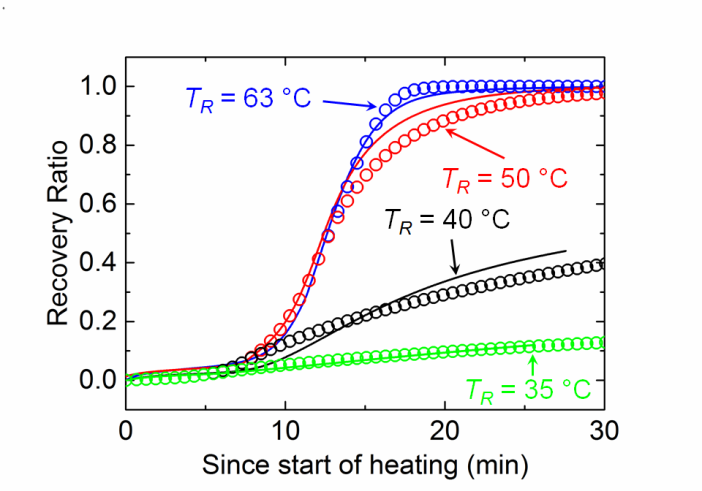


**a b**

**Figure S9| Model Predictions for the SMP made of 80% B+20% P750.** **a.** Strain-time plot SM behavior. **b.** Free recovery at different recovery temperatures (Solid lines represent experiments and discrete dots represent model predictions).

**S3** **Shape memory behavior of a printed spring**

**S3.1 Geometric description**

In order to investigate the nonlinear large deformation SM behavior of a printed spring, we conducted the SM cycling test of a printed spring with a representative segment shown in Figure S10a. The dimensions of the spring are given in Figure S10b and c. On the two ends of the spring, we printed two rings to facilitate the mounting to the testing machine. To mount the spring, two metal strings pass through the rings on the two ends of the spring and are fixed to the fiber clamps of the DMA machine (Figure S10d).


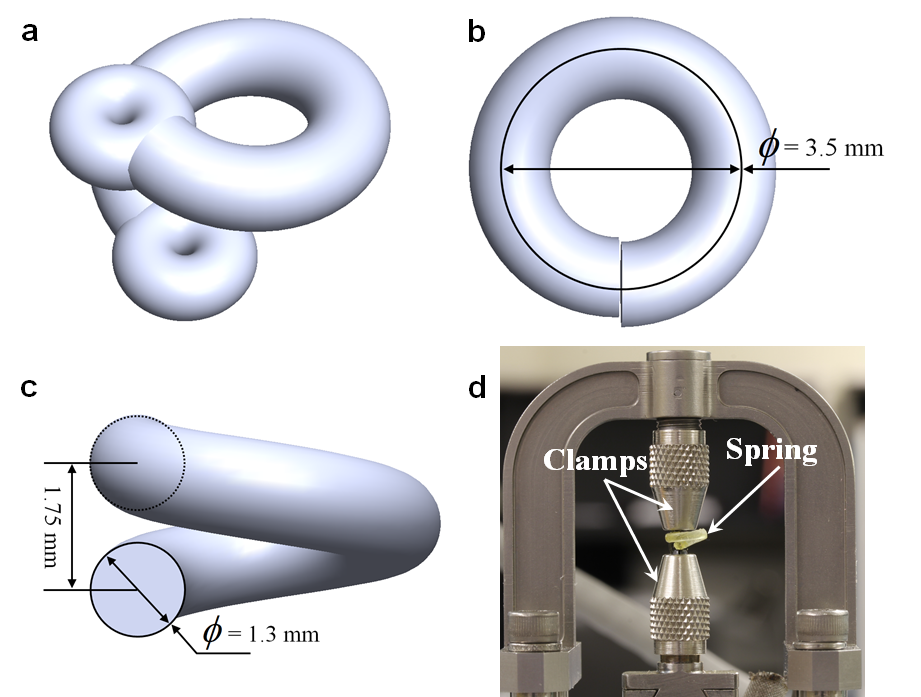


**Figure S10| Geometric description of one representative segment of a printed spring. a-c.** Isotropic, top and side of the spring design, respectively. **d.** The printed spring was mounted to the fiber clamp of the DMA machine.

**S3.2 Shape memory behavior of the printed spring**

We investigate the SM behavior of the printed spring by following the SM cycle shown in Figure S11a. The spring was stretched into a straight shape at 60 °C with a loading rate of 0.5 N/min to 0.8 N. Then, the temperature was gradually decreased to 20 °C with a cooling rate of 2 °C/min whilst the 0.8 N external load was maintained. After arrival at 20 °C, the temperature was first held for 2 min to reach the thermal equilibrium and then, the 0.8 N external was released within a few seconds. Finally, the temperature was increased to a recovery temperature (35 °C, 40 °C, 50 °C and 60 °C, respectively) with a heating rate of 5 °C/min to observe the free recovery behavior.

The measured displacement-time relation of the SM spring was presented in Figure S11b. The free recovery behavior is highly temperature dependent and can be described by the recovery ratio $R_{r}^{s}$ which is defined in the paper. By extracting the data from Figure S11b, we constructed Figure 4c in the paper presenting the temperature dependent free recovery behavior.

**S3.3 Finite element simulation of shape memory behavior**

Implementing the multi-branch introduced in S2.2 into user material subroutine (UMAT) in ABAQUS (Simulia, Providence, RI, USA) enables us to simulate this complex SM behavior of the printed spring. Figure S11c shows the origin shape of the spring which was meshed by 8-node linear brick, hybrid, reduced integration, hourglass control elements. All degrees of freedom of End A were fixed. At 60 °C, End B was loaded in the y-direction with a loading rate of 0.5 N/min to 0.8 N to stretch the spring. The external load was released after the temperature arrived at 20 °C. The temperature condition follows Figure S11a. Figure S11d compares the FE simulation with the experiment of the SM behavior. Overall, the FE simulation agrees the experiment well. At 60 °C, the Mises stress was ~3 MPa with the highest Mises stress in the main part of the spring. At 20 °C, the external load was deactivated, the Mises stress throughout the spring was reduced to zero and the straight shape was fixed. During the heating back to 60 °C, the spring was gradually recovered to the initial shape and Mises stress stayed at zero. Using this FE simulation, we also compared the force-displacement relation at 60 °C that is presented in Figure 4b of the paper and the recovery ratio over time during heating to different recovery temperatures in Figure 4c of the paper. Figure S11e also presents the simulated recovered shapes at different recovery temperatures which agree with the experiments in Figure 4c.


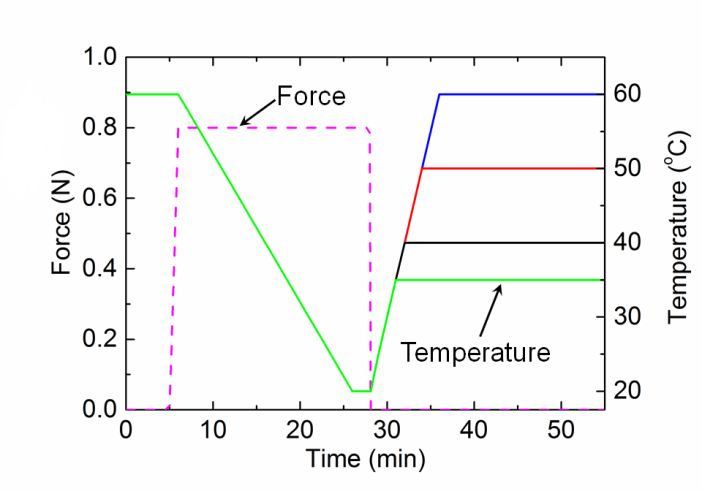

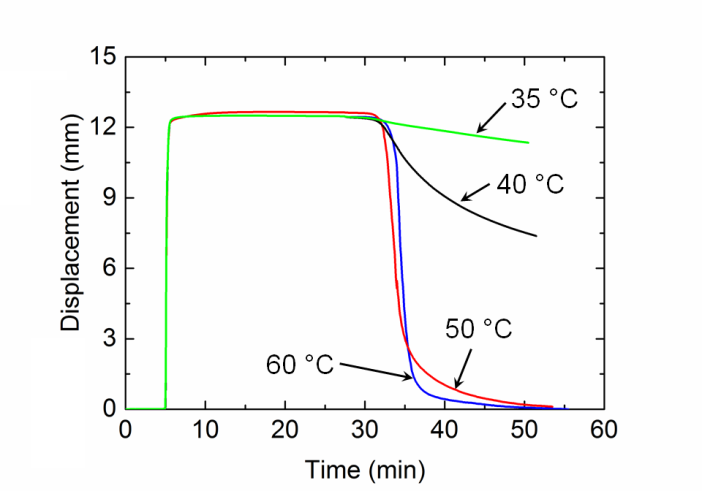


**a b**


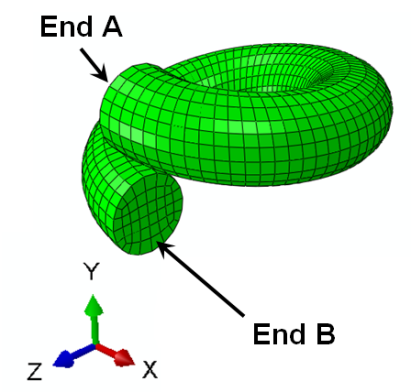

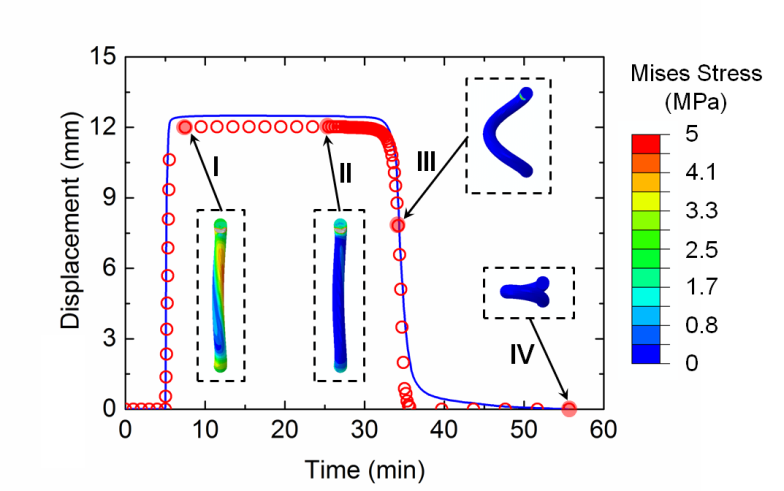


**c d**

*
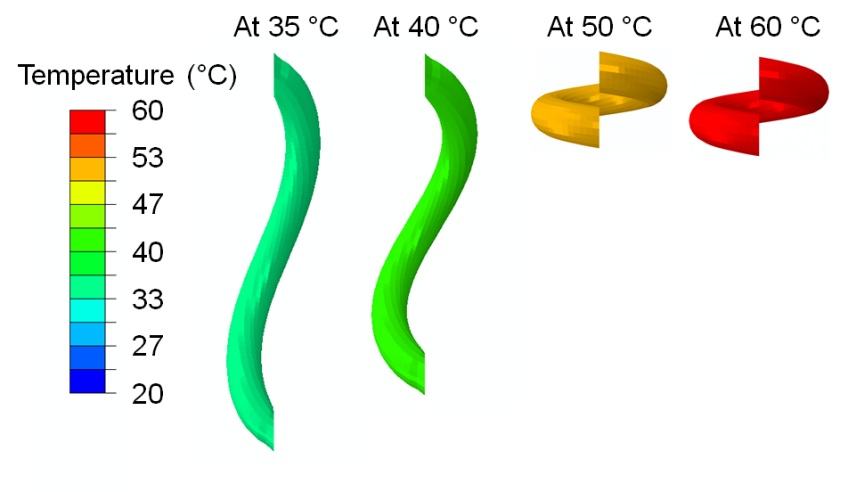
*

**e**

**Figure S11| Experiments and simulation of the printed spring. a.** Loading, temperature condition during the SM cycle. **b.** Displacement changes during the SM cycle at different recovery temperatures. **c.** Meshed spring and boundary condition. **d.** Comparison between FE simulation and experiment. **e.** Recovered shape at different recovery temperatures.

**S4** **Fabrication and simulation of multimaterial structures**

**S4.1 Multimaterial gripper**

In Figure S5b and c, the joints of multimaterial grippers were printed with 70% B +30% P550, and tips were printed with 50 % B + 50% P750 which is rubber at room temperature. We can choose other (meth)acrylate based polymers with different stiffness based on that of the object to realize a safe contact.

**S4.2 Simulation of multimaterial flower**

To simulate the sequential recovery of multimaterial flower, the flower model was which was meshed by 4-node linear brick, reduced integration, hourglass control elements. Based on List S6, the parameters of 90% +10% BPA were set to the inner petals, and the parameters of 80% +20% P750 were set to the outer petals. During simulation, we first folded the inner and outer petals at 70 °C, and decreased the temperature into 20 °C at a rate of 5 °C/min. After holding at 20 °C for 2 min, the external load was removed and the petals were fixed at the folded shape. During the heating, the outer petals were recovered at 50 °C, and the inner petals were recovered later at 70 °C.

**S5 Supplementary movies**

**Movie S1.** FE simulation of SM behaviour of the printed spring recovers into its original shape at different temperatures at 35 °C.

**Movie S2.** FE simulation of SM behaviour of the printed spring recovers into its original shape at different temperatures at 40 °C.

**Movie S3.** FE simulation of SM behaviour of the printed spring recovers into its original shape at different temperatures at 50 °C.

**Movie S4.** FE simulation of SM behaviour of the printed spring recovers into its original shape at different temperatures at 60 °C.

**Movie S5.** Free recovery of a shape memory Eiffel Tower.

**Movie S6.** The process of 3D printed multimaterial grippers to grab an object.

**Reference**
